# LABCG2, a New ABC Transporter Implicated in Phosphatidylserine Exposure, Is Involved in the Infectivity and Pathogenicity of *Leishmania*

**DOI:** 10.1371/journal.pntd.0002179

**Published:** 2013-04-25

**Authors:** Jenny Campos-Salinas, David León-Guerrero, Elena González-Rey, Mario Delgado, Santiago Castanys, José M. Pérez-Victoria, Francisco Gamarro

**Affiliations:** Instituto de Parasitología y Biomedicina “López-Neyra”, CSIC, (IPBLN-CSIC), Parque Tecnológico de Ciencias de la Salud, Armilla, Granada, Spain; Institut Pasteur, France

## Abstract

Leishmaniasis is a neglected disease produced by the intracellular protozoan parasite *Leishmania*. In the present study, we show that LABCG2, a new ATP-binding cassette half-transporter (ABCG subfamily) from *Leishmania*, is involved in parasite virulence. Down-regulation of LABCG2 function upon expression of an inactive mutant version of this half-transporter (LABCG2^K/M^) is shown to reduce the translocation of short-chain analogues of phosphatidylserine (PS). This dominant-negative phenotype is specific for the headgroup of the phospholipid, as the movement of phospholipid analogues of phosphatidylcholine, phosphatidylethanolamine or sphingomyelin is not affected. In addition, promastigotes expressing LABCG2^K/M^ expose less endogenous PS in the stationary phase than control parasites. Transient exposure of PS at the outer leaflet of the plasma membrane is known to be one of the mechanisms used by *Leishmania* to infect macrophages and to silence their immune response. Stationary phase/metacyclic promastigotes expressing LABCG2^K/M^ are less infective for macrophages and show decreased pathogenesis in a mouse model of cutaneous leishmaniasis. Thus, mice infected with parasites expressing LABCG2^K/M^ did not develop any lesion and showed significantly lower inflammation and parasite burden than mice infected with control parasites. Our results indicate that LABCG2 function is required for the externalization of PS in *Leishmania* promastigotes, a process that is involved in the virulence of the parasite.

## Author Summary

*Leishmania* is a protozoan parasite that infects human macrophages, producing the neglected tropical disease known as leishmaniasis. As is the case for apoptotic cells, transient exposure of phosphatidylserine (PS) on the surface of the parasite is required for macrophage engulfment and infection. Although the mechanism involved in this lipid translocation remains unknown, inhibition of PS exposure could therefore prove to be a novel way to combat this parasitic disease. Here, we have identified a new ABC transporter from *Leishmania*, namely LABCG2, as a protein involved in this process. The dominant-negative inhibition of LABCG2 showed that this transporter is required for the normal exposure of PS on the outer leaflet of the plasma membrane. This altered phenotype was subsequently found to be correlated with a deficient ability to infect mouse peritoneal macrophages. In addition, studies in a mouse model of cutaneous leishmaniasis showed that animals infected with parasites with down-regulated LABCG2 activity did not develop any lesions. Taken together, these results suggest a role for the *Leishmania* LABCG2 transporter in PS exposure, determining the virulence of the parasite.

## Introduction

Leishmaniasis is a neglected disease that is caused by different species of the protozoan parasite *Leishmania* [1]. This parasite has a digenetic life cycle in which it alternates between promastigote and amastigote stages. Inside the insect (sandfly) vector, non-infective promastigotes are transformed into infective parasites during metacyclogenesis. After the host is bitten by the sandfly, an intense neutrophilic infiltrate into the skin bite sites occurs accompanied by a significant recruitment of macrophages. Afterwards, *Leishmania* metacyclic promastigotes attach to neutrophils as the initial host cell, and are taken up by phagocytosis [2]. The uptake of infected neutrophils by macrophages is a mechanism for “silent” entry of parasites into macrophages, where they differentiate into the replicative amastigote forms that are responsible for maintenance and propagation of the infection in the phagolysosomal compartment of the mammal host [3], [4].

Phosphatidylserine (PS), a phospholipid (PL) normally asymmetrically confined on the inner leaflet of the plasma membrane of eukaryotic cells [5], seems to play a critical role in the infection of macrophages by *Leishmania* [6]–[9]. Indeed, PS exposure on the outer leaflet of the plasma membrane of apoptotic mammalian cells [10] constitutes the most central “eat-me” signal known for macrophages, which also “silent” its activity to avoid an inflammatory reaction [11]. In a process known as apoptotic mimicry, surface exposure of PS in *Leishmania* promastigotes and amastigotes is required for the infection of new mammalian cells [6], [7] and for down-regulation of the microbicidal activity of macrophages [8], [9], [12] by inhibiting their nitric oxide production and increasing IL-10 synthesis and TGFβ1 secretion [8], [13]. In addition, the well-characterized higher infectivity of the stationary phase promastigotes (metacyclic), as compared to the log phase promastigotes, is also due to the specific exposure of PS on their surface [14], among others factors including the lipophosphoglycan (LPG) or the phosphatidylinositol-anchored surface molecule gp63 [15]. Interestingly, it has been suggested that these PS-exposing promastigotes could be genuine apoptotic cells destined for death [12], [16] instead of apoptotosis-mimicking parasites. Indeed, their presence in the virulent inoculum, in an altruistic behaviour, provides survival advantages for the viable parasites and is necessary for progress of the disease [16]. Recently, it has been demonstrated that PS exposure by intracellular amastigotes of *L. amazonensis* is associated with a modified host inflammatory response, correlating with parasite infectivity and with clinical parameters of diffuse cutaneous leishmaniasis [17]. Thus, *Leishmania* parasites able to expose higher amounts of PS, induce a more severe and persistent human disease [17].

The plasma membrane PL asymmetry in eukaryotic cells is maintained due to the bidirectional transport of PL (flip-flop), which involves three protein-mediated activities [18]: i) flippases, which promote active inward-directed PL migration, mediated by aminophospholipid translocases (APT); ii) floppases, which are responsible for the active outward transport of PL from the cytoplasmic to the exoplasmic leaflet of the membrane, mediated by various ATP-binding cassette (ABC) transporters; and iii) scramblases, which are translocases that not require ATP to equilibrate the PL between the two membrane bilayers. PS externalization in apoptotic cells has been suggested to be due to i) a scramblases activity, enhanced by loss of the APT function [19]; and ii) to a higher activity of ABC efflux pumps such as ABCA1 [20]. Additionally, it has been suggested that PS is also delivered to the surface of lysosomes that fuse with the plasma membrane during apoptosis [21]. In the case of *Leishmania*, a decrease in the active out-to-in PS translocation, thus allowing ATP-independent PS movement, has also been suggested to be responsible for the loss of PL asymmetry [14]. However, disruption of the plasma membrane APT of *Leishmania* (LdMT) does not lead to an increased infectivity [22], [23]. In addition, although a scramblase activity has been described in *Leishmania*, its role in parasite infectivity remains to be elucidated [24]. The molecular basis of PS exposure in *Leishmania* therefore remains unsolved.

Functional ABC transporters consists of two homologous halves, each of which is composed of a transmembrane domain (TMD), which is involved in substrate binding and a cytosolic nucleotide binding domain (NBD), which hydrolyses ATP to provide the energy required for the transport [25]. The ATP sites are reconstituted upon dimerization of both NBDs, which pack together in a head-to-tail configuration to generate two ATP binding and hydrolysis sites between the conserved Walker A and B motifs of one NBD and the signature motif of the other [26]. ABC half-transporters with a single NBD therefore require homo-/heterodimerization to reconstitute the ATP sites. Members of the ABCA, ABCB and ABCG human subfamilies have been implicated in PL translocation [18], [27]. For example, human ABCG2 (BCRP/MXR/ABCP), a protein involved in multidrug resistance in cancer cells [28], [29], is responsible for enhanced exposure of PS at the plasma membrane of ABCG2 overexpressing cells due to increased outward PS transport [30]. Members of the ABCG subfamily of half-transporters have been identified in *Leishmania* [31], and three of these have already been functionally characterized. Thus, LABCG4 is localized at the plasma membrane of the parasite and is involved in the translocation of phosphatidylcholine (PC) analogues; it also confers resistance to alkyl phospholipids [32]. LABCG6 is also localized at the plasma membrane and is probably involved in PL trafficking as it reduces the accumulation of PL analogues of PC, phosphatidylethanolamine (PE) and PS [33], and confers resistance to camptothecin [34], miltefosine and sitamaquine [33]. LABCG5, in contrast, is not involved in the translocation of PL at the plasma membrane or drug resistance but participates in salvage of the heme released after the breakdown of internalized haemoglobin [35]. Additionally, it has been reported that other *Leishmania* ABC transporters such as LABCB4 [36], LABCA1 [37] and LABCA2 [38] are involved in PL translocation.

The aim of our work was to study the functionality of the transporter LABCG2 from *Leishmania*, specifically its involvement in PS translocation and its implication in parasite virulence. The results show that down-regulation of LABCG2 produces a defect in the exposure of endogenous PS at the external surface of the parasite, and that this defect correlates with a significant decrease in the ability of these parasites to infect mouse peritoneal macrophages and to produce pathology in a mouse model of cutaneous leishmaniasis.

## Materials and Methods

### Materials

3-(4,5-Dimethylthiazol-2-yl)-2,5diphenyltetrazolium bromide (MTT), PMSF (phenylmethylsulfonyl fluoride), DFP (diisopropylfluorophosphate), monoclonal anti α-tubulin, and amphotericin B were obtained from Sigma Chemical Company (St. Louis, USA). Anti-histone H2A was courtesy of Dr. Stephen M. Beverley (Washington University, School of Medicine, St. Louis, Missouri, USA). Polyclonal anti-GFP antibody was from Rockland Company. Mouse monoclonal anti-gp63 was from Life Span BioSciences. Polyclonal antisera against metacyclic marker protein HASPB was a kind gift from Dr. Deborah F. Smith (University of York, UK). The fluorescent analogues 1-palmitoyl-2-[6-(7-nitrobenz-2-oxa-1,3-diazol-4-yl)amino]hexanoyl*-sn*-glycero-3-phosphocholine (NBD-PC), -phosphoethanolamine (NBD-PE), -phosphoserine (NBD-PS) and 6-(7-nitrobenz-2-oxa-1,3-diazol-4-yl)amino-hexanoyl-sphingosine-1 phosphocholine (NBD-sphingomyelin; NBD-SM) were purchased from Avanti Polar Lipids (Birmingham, AL, USA). Annexin V-Alexa 488, FM4-64, concanavalin A-Alexa Red, MitoTracker Deep Red 633, Cell Tracker TM Green and DAPI were from Molecular Probes (Invitrogen, Carlsbad, CA). Ro-peptide (Ro09-0198), a tetracyclic peptide antibiotic, was kindly provided by Dr. Masato Umeda (The Tokyo Metropolitan Institute of Medical Science, Japan). Papuamide B (Flintbox, LynseyHuxham), a novel depsipeptide obtained from extracts of marine sponges, was kindly provided by Dr. Thomas Gunther Pomorski and Dr. Rosa López (Department of Plan Biology and Biotechnology, University of Copenhagen, Denmark). Peanut agglutinin (PNA) and fluorescein-conjugated ricin agglutinin was purchased from Vector (Burlingame, CA). The plasmids pXG-GFP+2′ and pXG-'GFP, which can be used to express GFP fusion proteins in *Leishmania* with GFP at either the N- or the C-terminus, respectively, were kindly provided by Dr. Stephen M. Beverley.

### *Leishmania* strains and cell cultures

Promastigote forms of *Leishmania major* clone V1 (MHOM/IL/80/Friedlin), *Leishmania infantum* (MHOM/ES/1993/BCN-99) and *Leishmania donovani* (MHOM/ET/67/HU3) were maintained *in vitro* at 28°C in modified RPMI-1640 medium (Invitrogen, Carlsbad, CA) supplemented with 20% heat-inactivated foetal bovine serum (hiFBS, Invitrogen), as described previously [37], [38]. To determine parasite sensitivity to the toxic peptides papuamide B and Ro-peptide, and to amphotericin B, 10^6^/mL parasites were incubated in RPMI-1640 containing different concentrations of peptides and the parasite viability determined by MTT analysis after 72 h, as described previously [39].

### DNA constructs and cell transfection

*LABCG2* (GeneDB-*L. major*, Accession Code LmjF06.0090) was isolated from the genomic DNA of *L. major* by PCR using sense (5′- ATATCGCTGTCTCTGCGTCG) and antisense (5′- GGCAAACACACAGAGCGATG) primers. The nucleotide sequences were determined automatically as previously described [40]. To obtain parasites expressing non-functional LABCG2, a mutation was introduced in the Walker A motif of the ATP binding domain, replacing lysine 108 with a methionine (K108M) using the QuikChange XL Site-Directed Mutagenesis kit (Stratagene, La Jolla, CA). The resulting mutated gene *LABCG2^K108M^* (*LABCG2^K/M^*) was cloned into the *Leishmania* expression vector pUCNeoPlus [37]. The vector pXG-GFP+2′ was used to create GFP- LABCG2 and -LABCG2^K/M^ versions with GFP fusions at N-terminus [41]. The LABCG2 and LABCG2^K/M^ open reading frame for these N-terminus tagged versions was amplified by PCR using sense (5′-GCGGCCGCATGCCCCCTCCCGCAGCAACACGTGC) and antisense (5′-GCGGCCGCTCATGCCGTTCTCGCACAGCTCGCCA) primers. For the C-terminus tagged versions, LABCG2 and LABCG2^K/M^ were cloned in a pXG-'GFP+ using sense (5′-ACCGGTATGCCCCCTCCCGCAGCAACACGTGC) and antisense (5′- GATATCTGCCGTTCTCGCACAGCTCGCCACGG) primers. Promastigotes of *L. major* were transfected with the different constructs and selected for G-418 resistance as described previously [42].

### Gene expression analysis

Total RNA was prepared from control (empty vector) and LABCG2^K/M^ expressing promastigotes using the total RNA isolation kit (Roche Biochemicals). cDNA was synthesized from 60 ng of total RNA using Superscript II TM RNaseH Reverse Transcriptase (Invitrogen) and oligo (dT)12–18 primers (Invitrogen) following the manufacturer's instructions. Semi-quantitative PCR was performed with 50 µL aliquots using 50 pmol each of sense and antisense primers corresponding to LABCG2 and LmGAPDH using the following profile: initial denaturation at 95°C for 5 min followed by 25 cycles with denaturation at 95°C for 1 min, annealing at 54°C for 30 s and extension at 68°C for 35 s, with a final extension of 5 min.

### Fluorescence microscopy of *Leishmania* promastigotes

For endosome/lysosomal labelling, 10^7^ stationary-phase promastigotes obtained after 4 day culture were incubated in 1 mL of RPMI 1640 medium containing 50 µg/mL of concanavalin A-Alexa Red for 2 h at 28°C or with 1 µM FM4-64 for 30 min at 28°C or 4°C. For mitochondrial labelling, 10^7^ stationary-phase promastigotes were stained with 50 nM MitoTracker Deep Red 633 for 30 min at 28°C and then washed in ice-cold phosphate-buffered saline (PBS; 1.2 mM KH_2_PO_4_, 8.1 mM Na_2_HPO_4_, 130 mM NaCl and 2.6 mM KCl adjusted to pH 7). Parasites were fixed for 30 min at 4°C with 2% paraformaldehyde and then observed under a microscope. Images (one stack) were acquired using an Olympus IX81 microscope and deconvolved using Huygens Professional.

### Analysis of fluorescent PL uptake

The NBD-phospholipid accumulation was determined by flow cytometry as described previously [43]. Briefly, stationary-phase promastigotes (10^7^/mL) were incubated in HPMI buffer (20 mM HEPES, 132 mM NaCl, 3.5 mM KCl, 0.5 mM MgCl_2_, 5 mM glucose, 1 mM CaCl_2_, pH 7.4) supplemented with 0.3% (w/v) BSA for 30 min at 28°C, then labelled with 10 µM NBD-PC, 10 µM NBD-PE, 10 µM NBD-SM or 30 µM NBD-PS for 30 min at 28°C. HPMI was supplemented with either 500 µM PMSF or 5 mM DFP to block the catabolism of NBD-lipids [43]. Parasites were washed twice with ice-cold PBS, supplemented with 0.3% BSA and resuspended in PBS for flow cytometry analysis, using a Beckton Dickinson FACScan (San José, CA) equipped with an argon laser operating at 488 nm.

### Measurement of NBD-PS outward transport in *L. major* lines

To measure the NBD-PS outward transport from the cytoplasmic to the exoplasmic leaflet, *Leishmania* stationary-phase promastigotes (10^7^/ml) were labeled with 30 µM (control parasites) or 15 µM (LABCG2^K/M^) NBD-PS for 30 min at 28°C in HPMI buffer (20 mM HEPES, 132 mM NaCl, 3.5 mM KCl, 0.5 mM MgCl_2_, 5 mM glucose, 1 mM CaCl_2_, pH 7.4) supplemented with 0.1% (w/v) BSA to allow that inward movement of the NBD analogue was equally, as previously described [30]. Afterwards, NBD-PS remaining on the cell surface was extracted twice by incubation with 2% (w/v) BSA in HPMI (supplemented with 5 mM glucose and 500 µM PMSF) for 5 min on ice. Before starting the outward transport assay, the medium was removed and parasites were washed with ice-cold PBS. For *t* = 0 min, the cells were resuspended in HPMI (supplemented with 2% BSA, 5 mM glucose and 500 µM PMSF). Time dependent outward transport was monitored at 28°C at different time points (5, 15, 30, 60 min) in the supernatants and the samples were analyzed by SLM-Aminco 8000C spectrofluorimeter.

### Annexin V- binding assay

*Leishmania* promastigotes were harvested in RPMI-1640 and centrifuged at 2500× *g* for 10 min at 4°C. The cells were washed with Annexin V-binding buffer (20 mM HEPES, 132 mM NaCl, 3.5 mM KCl, 5 mM CaCl_2_ and 0.5 mM MgCl_2_, pH 7.4 and 10 mM glucose), then resuspended in the same buffer and incubated with Annexin V–Alexa 488 (1/20 dilution; at the concentration indicated by the manufacturer) at 4°C for 15 min. The parasites were subsequently labelled with propidium iodide (0.4 µg/ml) and the mixture incubated for 5 min at 4°C. The cells were washed for 1 min at 2500× *g* and 4°C, and resuspended to a cell density of 4×10^6^ cells/mL. Controls measurements in the absence of calcium were included using Annexin V–Alexa 488 plus 8 mM EGTA. Cellular fluorescence was quantified by scanning the emission in a FACSCalibur and analysed using the Cell Quest Pro software application. A total of 10,000 events were harvested from each sample. The control cells were incubated in Annexin V-binding buffer alone, without Annexin V–Alexa 488, under identical conditions.

### *In vitro* infection of mouse peritoneal macrophages

Peritoneal macrophages from BALB/c mice (Charles River Ltd.) were harvested by lavage with ice-cold RPMI 1640 medium, plated at a density of 5×10^5^ macrophages/well in RPMI-1640 medium plus 10% hiFBS in 24-well plates provided with glass coverslips (22 mm^2^) and allowed to adhere for 4 h at 37°C under 5% CO_2_, as described previously [7], [8]. The adherent macrophages were infected at 35°C with stationary-phase promastigotes of control and LABCG2^K/M^-expressing *L. major* parasites with or without Annexin V (0.05 µg/µl×10^7^promastigotes), at a parasite-to-cell ratio of 5∶1 in RPMI-1640 medium supplemented with 5% hiFBS. After 4 h of infection, unphagocytosed parasites were removed by washing with serum-free medium. The infected macrophages were further incubated in RPMI 1640 medium supplemented with 10% hiFBS for 24 h at 37°C in a 5% CO_2_ atmosphere. Following incubation, the cultures were fixed with 2% paraformaldehyde/glucose, stained with DAPI and the rate of infected macrophage analyzed using images acquired with an Olympus IX81 microscope as described previously [44]. Parasites were quantified using a cell counter provide with the Image J software (http://rsb.info.nih.gov/ij/). The images were deconvolved using Huygens Professional from Scientific Volume Imaging (http://www.svi.nl). The percentage of macrophage infection was calculated by dividing the number of infected macrophages by the number of counted macrophages. The mean number of amastigotes per infected macrophage was determined by dividing the total number of amastigotes counted by the number of infected macrophages. Three independent experiments were performed with duplicates.

### *Leishmania* binding assays

The interactions between *L. major* stationary-phase promastigotes and mouse peritoneal macrophages were measured as previously described with some modifications [22]. Mouse peritoneal macrophages (5×10^5^/well), maintained at 37°C with 5% CO_2_ in RPMI 1640 medium supplemented with 10% of hiFBS, were labeled with 5 µM FM4-64 for 30 min at RT in RPMI 1640 medium. LABCG2^K/M^ and control *L. major* promastigotes (10^7^/ml) in stationary phase were labeled with 1 µM Cell Tracker TM Green for 40 min at 28°C in RPMI 1640 medium. Then, parasites and peritoneal macrophages were washed four times in RPMI 1640 medium and finally resuspended in RPMI 1640 medium supplemented with 5% of hiFBS. Binding assays were performed using a parasite∶macrophage ratio of 5∶1. Promastigote forms of *L. major* lines were added to the monolayer cells. After 4 h incubation at 37°C, unbound promastigotes were removed by thorough washing. The monolayers and bound promastigotes were analysed by a Confocal Leyca SP5 microscopy. All the experiments were done in triplicate.

### LPG and gp63 surface expression analysis

Expression analysis of the surface molecule LPG was performed as described previously [45]. Thus, stationary-phase promastigotes (10^7^/mL) were washed twice with PBS and incubated with 5 µg/mL fluorescein-conjugated ricin agglutinin in PBS for 10 min at 28°C, then washed with PBS and analyzed by flow cytometry using a FACSCalibur (Beckton Dickinson). For quantification of cell surface gp63, parasites were incubated with a 1∶500 dilution of a mouse monoclonal anti-gp63 on ice. The cells were subsequently washed with PBS supplemented with 0.1% BSA and fixed at 4°C in 2% paraformaldehyde for 20 min, then washed again and incubated at room temperature with a 1∶500 dilution of FITC fluorescein isothiocyanate-labeled goat anti-mouse immunoglobulin G (Sigma). These cells were washed three times with PBS-0.1% BSA and the parasite-associated fluorescence was analyzed by flow cytometry using a FACSCalibur.

### Metacyclic purification assay

Metacyclic promastigotes were isolated from stationary cultures of *L. major* promastigotes by negative selection using a previously described peanut agglutinin (PNA) methodology [46]. Briefly, stationary-phase promastigotes were collected after culture for 4 days, washed with PBS and then incubated with 100 µg/mL of PNA. After incubation for 10 min at room temperature, cells were separated by centrifugation at 500× *g* for 10 min. The non-agglutinated promastigotes (metacyclic) collected in the supernatant were washed twice with PBS and resuspended in PBS for further experiments.

### Western blot analysis

Proteins from whole stationary-phase promastigotes (10^7^/well) were resolved in 10% SDS-PAGE and electroblotted onto PVDF membranes. Western blot analysis was performed as described previously [47], using a polyclonal antibody against metacyclic promastigote protein HASPB (1∶2000) or GFP (1∶5000; Invitrogen), followed by detection with a horseradish peroxidase-conjugated secondary goat anti-rabbit IgG (1∶5000; Dako Denmark) antibody. Monoclonal antibodies against H2A histone or α-tubulin (1∶5000 or 1∶10000; Sigma-Aldrich) were used to confirm equivalent protein loading. Detection was carried out by enhanced chemiluminescence reaction using the ECL kit (Amersham).

### Analysis of *in vivo* infection

Six-week-old female BALB/c mice were purchased from Charles River Breeding Laboratories and maintained in the Animal Facility Service of our Institute under pathogen-free conditions. Animals (10 mice/group) were injected subcutaneously in their left hind footpads with 10^4^ *L. major* purified metacyclic promastigotes resuspended in PBS, as described above. Disease progression was monitored by measuring the inflammation edema and the area of the cutaneous lesion of the infected footpad using a digital caliper (Mitutoyo, Japan), in comparison with the values obtained in the uninfected contralateral footpad. Parasite burdens in target tissues were determined from the presence of amastigotes isolated from footpad, spleen and lymph nodes at week five post-infection, after tissue homogeneization and culture in promastigote culture medium, using a limiting dilution assay, as described previously [48].

### Ethics statement

All experiments were performed according to the National/EU Guidelines for the Care and Use of Laboratory Animals in Research and the approval of the Ethics Committee of the Spanish National Research Council (CSIC, file CEA-213-1-11).

### Statistical analysis

Statistical comparisons between groups were performed using Student's *t* test. Differences were considered significant at a level *p*<0.05.

## Results

### Sequence features of LABCG2 and generation of a *Leishmania* line expressing an inactive version of the protein

LABCG2 (GeneDB-*L. major*, accession code LmjF06.0090) has two additional tandem imperfect repeats in chromosome 6 of *Leishmania* (LABCG1, accession code LmjF06.0080, and LABCG3, accession code LmjF06.0100) [31]. LABCG1, LABCG2 and LABCG3 are “half-transporters” with a single NBD and a single TMD localized at their C-terminus (Appendix in Supporting information, Fig. S1A). *LABCG1* and *LABCG2* codes for a 657 and 663 amino acid protein, with a predicted molecular weight of approximately 73.4 and 74.0 kDa, respectively. LABCG1 and LABCG2 are almost identical (95.7% of identity). LABCG3 protein is truncated, with Walker A and several transmembrane segments being absent (Appendix in Supporting information, Fig. S1B). LABCG2 shares 19.5% amino acid identity with human ABCG1, 24.6% with human ABCG2 and 27.3% with the White protein from *Drosophila* [35].

The dimerization requirement for ABC half-transporters (such as LABCG2) to become functional led us to test a dominant-negative approach to down-regulate LABCG2 function, as recently described for *Leishmania* LABCG5 [35]. To this end, we first mutated in LABCG2 the lysine residue inside the Walker A motif (108 position), which is known to be critical for ATP hydrolysis in ABC transporters [35], [49], to a methionine (K108M). The expression of other ABCG half-transporters with a similar K/M substitution produces a dominant-negative inhibition in the wild-type transporters [35], [50]. The resulting construct was transfected into *L. major* and the expression of LABCG2^K108M^ (LABCG2^K/M^) was verified by RT-PCR (Appendix in Supporting information, Fig. S2A). In contrast to the phenotype observed after LABCG5^K/M^ expression [35], parasites transfected with LABCG2^K/M^ grew at normal rates (Appendix in Supporting information, Fig. S2B).

### Subcellular localization of LABCG2

To study the localization of LABCG2, we fused LABCG2 and LABCG2^K/M^ with an N-terminal GFP-tag. These constructs were transfected into *L. major* promastigotes and expression of these proteins determined by Western-blot analysis of whole parasite lysates. As expected, a band corresponding to GFP-LABCG2 was observed at around 100 kDa (Appendix in Supporting information, Fig. S3A). Additional higher molecular weight signals could correspond to dimeric forms of the protein, as described for LABCG5 [35], whereas the lower bands probably correspond to degraded proteins.

**Figure 1:**
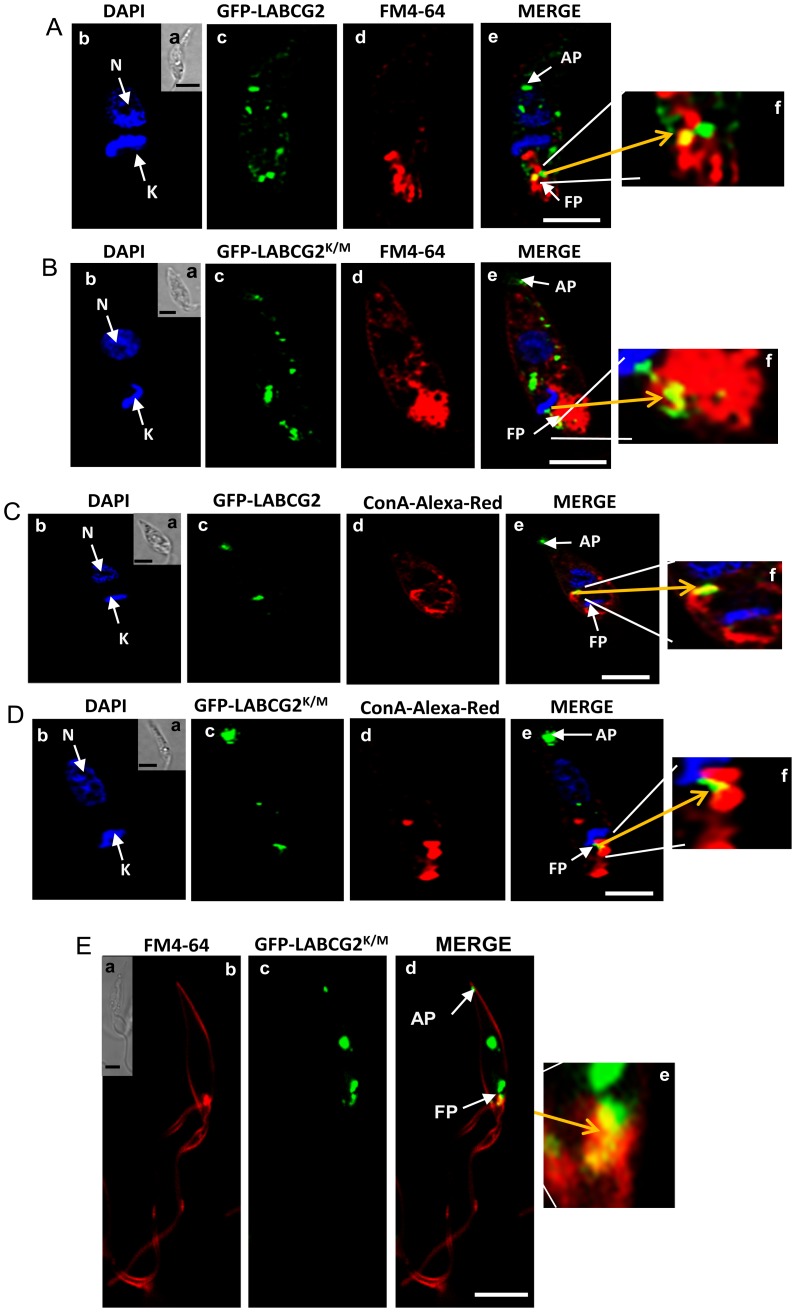
LABCG2 localizes to the intracellular vesicles of *Leishmania* parasites. *L. major* stationary promastigotes transfected with GFP-LABCG2 (A and C) and GFP-LABCG2^K/M^ (B and D) were incubated at 28°C with 1 µM FM4-64 (A and B) and 50 µg/mL concanavalin A-Alexa Red (C and D) for 30 min and 120 min, respectively. (E) LABCG2^K/M^ localizes into the flagellar pocket of *Leishmania* parasites. *L. major* stationary promastigotes transfected with GFP-LABCG2^K/M^ were incubated with 1 µM FM4-64 for 30 min at 4°C. The parasites were fixed for 10 min in 2% with paraformaldehyde at 4°C. (*a*) Nomarski images of *b*, *c*, *d* and *e*. Representative co-localization sites (*f*) are indicated by yellow arrows in the merged images. Scale bar: 5 µm. N (nucleus) and K (kinetoplast) are stained with DAPI. FP: flagellar pocket; AP: aflagellar pole. The figure illustrates a representative parasite of a total population of parasites with a similar fluorescence pattern.

Fluorescence microscopy studies showed that GFP-LABCG2 partially overlap with the endosomal markers FM4-64 [51] and concanavalin A [52] in the stationary growth phase promastigotes, which is depicted in representative micrographs in Fig. 1A and 1C. In contrast, the stained vesicular structures do not co-localize with the mitochondrial marker MitoTracker (data not shown). The localization pattern of the protein does not change when GFP-LABCG2^K/M^ was expressed in *Leishmania* parasites (Fig. 1B and 1D). To evaluate whether GFP-LABCG2 was also localized in the flagellar pocket, the sole site for endo-/exocytosis in *Leishmania*, we subsequently performed the co-localization experiments with FM4-64 at 4°C to block its vesicular trafficking. The results showed that GFP-LABCG2^K/M^ co-localizes in the flagellar pocket of the stationary-phase promastigotes (Fig. 1E, yellow arrows). Another part of the GFP-LABCG2^K/M^ pool was detected in intracellular vesicles localized in the apical part of the cell, at the tip of the aflagellar pole of the parasite (Fig. 1E), a site that is known to be involved in the interaction with host cells [53]. GFP-tagged LABCG2 protein at the COOH-terminal showed a similar pattern of localization (Appendix in Supporting information, Fig. S3B), but its expression was unstable after few culture passages, thus suggesting that the C-terminal TMD region is critical for maintaining LABCG2 stability. Overall, these studies suggest that LABCG2 localizes to intracellular vesicles of the endosomal pathway, at the flagellar pocket and at the aflagellar pole of *Leishmania*.

### LABCG2 is involved in the exposure of PS on the outer surface of *Leishmania*

**Figure 2:**
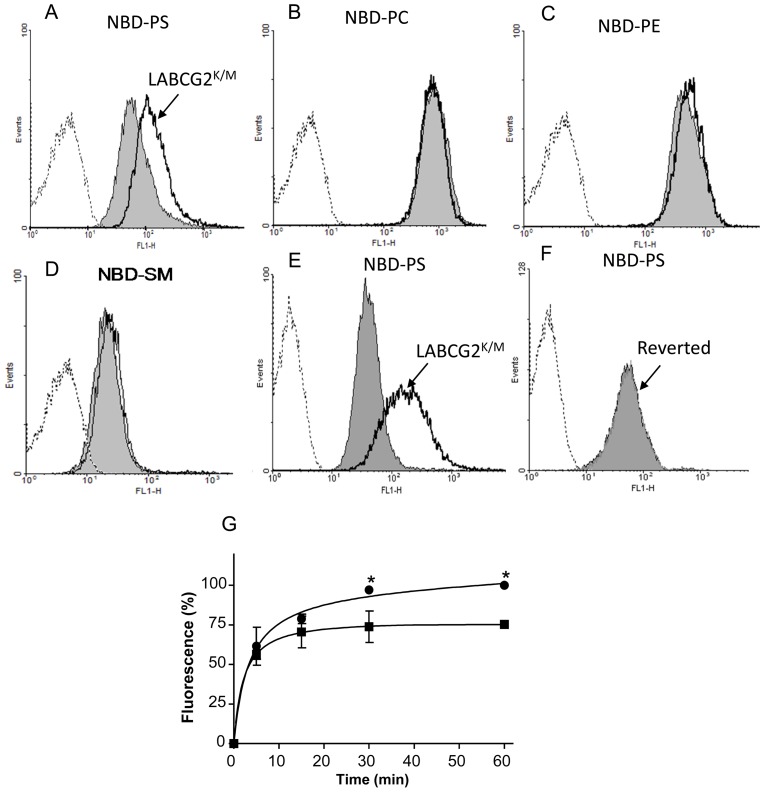
Fluorescent PL accumulation in *Leishmania* parasites. Stationary promastigotes of *Leishmania* were incubated with the fluorescent PL analogues NBD-PS (A), NBD-PC (B), NBD-PE (C) or NBD-SM (D) for 30 min at 28°C. After washing and back-exchange with BSA, cell-associated fluorescence was measured by flow cytometry analysis. The grey histogram represents control cells transfected with the empty vector, the uncoloured histogram represents parasites expressing LABCG2^K/M^ and the dotted histogram represents non-labelled cells. (E) NBD-PS accumulation in *Leishmania* lines after a second transfection event. The reverted line (F), which was maintained for 3 months without drug pressure, was also included. The histograms correspond to a representative experiment from three independent experiments. (G) Outward transport of NBD-PS in *Leishmania* parasites. Stationary promastigotes of control (black circles) and LABCG2^K/M^ (black squares) *Leishmania* lines were incubated with the fluorescent analogue NBD-PS for 30 min at 28°C. After washing and back-exchange with BSA, cells were incubated for different time points in a free NBD-PS medium containing BSA and the fluorescence of the supernatant was measured by spectrofluorimetry. Results represent the means ± SD of four independent duplicated experiments. * *P*<0.05 vs. control parasites.

To study the possible role of LABCG2 in PL transport, we first investigated the accumulation level of fluorescent short-chain PL analogues by flow cytometry. Thus, stationary-phase *L. major* promastigotes transfected with the empty vector (control) or the vector containing LABCG2^K/M^ (LABCG2^K/M^ parasites) were incubated with NBD-PE, -PC, -PS and -SM, and the cell-associated fluorescence analyzed by flow cytometry after back-exchange with BSA to extract the NBD-PL located in the outer plasma membrane leaflet. Under these conditions, accumulation of NBD-PS by the LABCG2^K/M^ parasites was significantly higher than that observed for control cells (4.2 fold, n = 12, *p*<0.05; Fig. 2A). In contrast, no significant differences were observed for NBD-PC, NBD-PE and NBD-SM accumulation between control and LABCG2^K/M^ parasites (Fig. 2B–D). The change in NBD-PS accumulation was not due to differences in endocytosis, as the internalization of NBD-SM, which is taken up by this process, was not affected by the functionality of LABCG2 (Fig. 2D). The above results were validated in a second transfection event with LABCG2^K/M^ (Fig. 2E). To verify that the higher NBD-PS accumulation observed was due to the dominant-negative inhibition of LABCG2 activity, LABCG2^K/M^ parasites were cured for the plasmid pUCNeoplusLABCG2^K/M^ (reverted line) by culturing the parasites in the absence of plasmid drug selection pressure for three months. This reverted line showed a similar NBD-PS accumulation to the control line (Fig. 2F). We subsequently tested whether LABCG2 was involved in NBD-PS internalization in two other *Leishmania* species (*L. infantum* and *L. donovani*). Down-regulation of LABCG2 function was also assayed by expressing LABCG2^K/M^ and a similar phenotype was observed in these *Leishmania* species (Appendix in Supporting information, Fig. S4A and B). To confirm that the higher accumulation of NBD-PS in the LABCG2^K/M^ parasites was due to a reduced floppase activity, we measured the outward translocation of NBD-PS from the cytoplasmic to the exoplasmic leaflet of the plasma membrane in both *Leishmania* lines. Thus, control and LABCG2^K/M^ *L. major* promastigotes were loaded under conditions that yielded similar amounts of intracellular NBD-PS after the back-exchange step. Then, parasites were maintained in probe-free culture medium containing BSA and the amount of NBD-PS extracted from the external side of the plasma membrane was measured at different time points. The results showed that the outward translocation of NBD-PS was higher in control versus LABCG2^K/M^ promastigotes (Fig. 2G) suggesting a PS floppase activity of LABCG2.

**Figure 3:**
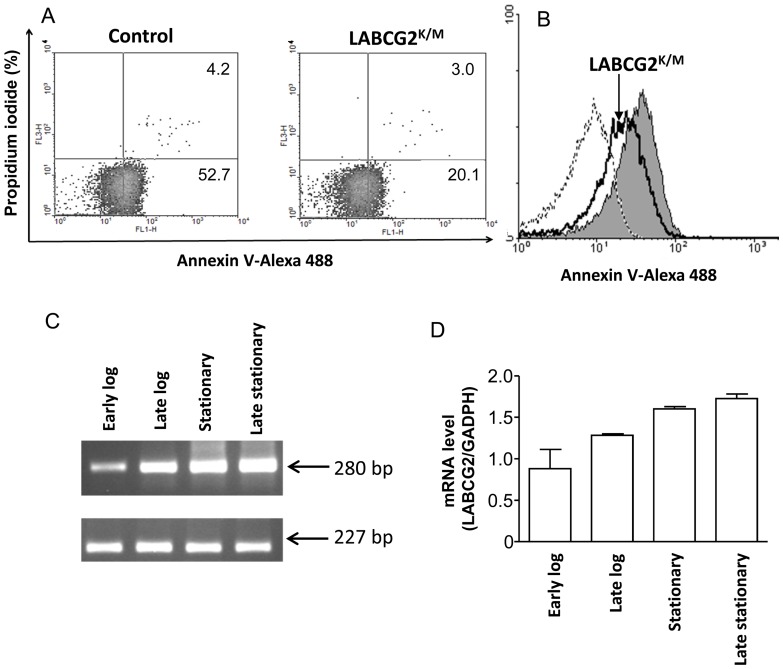
The externalization of endogenous PS by stationary *Leishmania* promastigotes depends on LABCG2 function. (A) PS exposure at the outer leaflet of the parasite plasma membrane was analyzed by flow cytometry using Annexin V–Alexa 488 as described in Materials and Methods. The lower right quadrant in the density plots represents the percentage of Annexin V ^positive^/Propidium iodide ^negative^ in control or LABCG2^K/M^ parasites. Markers were placed using non-stained parasites. (B) Density of PS molecules (GeoMean) on the cell surface. The grey histogram represents control cells transfected with the empty vector, the uncoloured histogram represents parasites expressing LABCG2^K/M^ and the dotted histogram represents non-labelled cells. The results shown are representative of three independent duplicated experiments. (C and D) Gene expression analysis of LABCG2 from *L. major* control determined by RT-PCR analysis through the different growth phases of *Leishmania* parasites: early log (day 2), late log (day 3), stationary (day 4) and late stationary phase (day 5). RT-PCR was carried out for 35 cycles using RNA isolated from the above parasites and the products were run in 2% agarose gel as described in Materials and Methods. Lower imagine in C shows the expression of *GADPH* used as internal loading control. The arrows indicate amplified 280 bp *LABCG2* fragment and 227 bp *GADPH* fragment. Lower imagine in D shows the mRNA level of LABCG2 normalized with GADPH in different points of the growth curve. The results shown are the means of three independent experiments ± SD.

**Figure 4:**
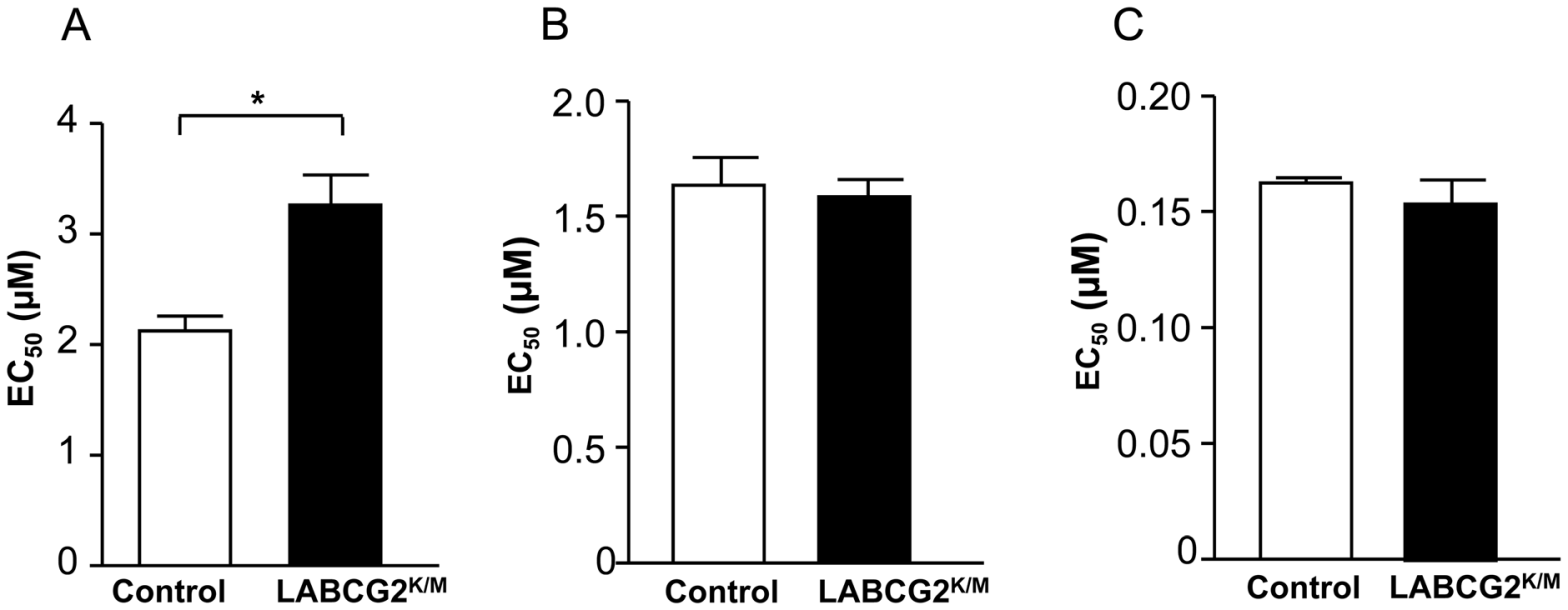
*Leishmania* LABCG2^K/M^ parasites present resistance to papuamide B. Sensitivity of control and LABCG2^K/M^ parasites to the PS-binding peptide papuamide B (A), PE-binding peptide Ro09-0198 (B) and amphotericin B (C). Logarithmic-phase promastigotes were diluted to 10^6^/mL in RPMI 10% hiFBS containing different concentrations of these peptides; after 72 h, cell viability was analysed by a MTT analysis as described in Materials and Methods. Results represent the means ± SD of four independent duplicated experiments. * *P*<0.05 vs. control parasites.

Next, we studied the translocation of endogenous, long-chain PS labelling control and LABCG2^K/M^ stationary growth phase promastigotes with Annexin V-Alexa Fluor 488. This probe binds PS exposed in mammalian apoptotic cells [8] and *Leishmania* promastigotes in a calcium dependent manner [16] (Appendix in Supporting information, Fig. S5). Quantitative analysis by flow cytometer (Fig. 3A) showed that LABCG2^K/M^ parasites presented a significantly reduced exposure of PS in the outer leaflet of the plasma membrane compared with control cells (20.1% vs. 52.7% of Annexin V^ positive^/propidium iodide^ negative^, respectively, *p*<0.05). Additionally, we determined that the density of PS molecules on the cell surface is significantly higher in the control (53.35±6.12) than in the LABCG2^K/M^ parasites (38.10±3.49), as measured by the mean fluorescence intensity (Fig. 3B); however, control and LABCG2^K/M^ log phase parasites showed a low and similar Annexin V-binding (10.97% vs. 11.06% of Annexin V^ positive^/propidium iodide^ negative^, respectively, *p*<0.05; data not shown). These results were supported by RT-PCR analysis of expression of LABCG2 through the life cycle of *L major* that shows higher expression of LABCG2 in stationary growth phase/metacyclic promastigotes versus log phase parasites (Fig. 3C and 3D). To validate that the differences in Annexin V-Alexa Fluor 488 labelling were due to a defect in PS exposure, we tested the sensitivity of control and LABCG2^K/M^ parasites to papuamide B, a pore-forming cytolytic peptide that specifically binds to PS at the external surface of the plasma membrane [54]. The results showed that LABCG2^K/M^ parasites were less sensitive to papuamide B than controls (EC_50_ = 3.26±0.67 µM vs. EC_50_ = 2.12±0.33 µM, *p*<0.05, respectively) (Fig. 4A). As a control experiment, we tested the sensitivity of both lines to Ro-peptide, which strictly recognizes PE residues at the external surface of biological membranes [55]. The results of this study indicated that there were no differences in sensitivity between LABCG2^K/M^ and control parasites (EC_50_ = 1.58±0.09 µM vs. EC_50_ = 1.64±0.12 µM, respectively) (Fig. 4B). These results are in agreement with the absence of alterations in NBD-PE translocation in LABCG2^K/M^ parasites. Several ABCG transporters have been implicated in sterol transport [56]. Thus, differences in papuamide B sensitivity might be indirectly caused by a general change in membrane lipid organization in LABCG2^K/M^ parasites. We study this possibility by analyzing both sensitivity of control and LABCG2^K/M^ parasites to amphotericin B; the results shown that there were no significant differences in sensitivity of both lines to amphotericin B (Fig. 4C), suggesting that LABCG2 does not contribute greatly in the distribution of sterols into the plasma membrane. Finally, we confirmed that the parasites expressing GFP-LABCG2^K/M^ used for the subcellular localization analysis also maintained their papuamide B resistance phenotype (data not shown).

### Down-regulation of LABCG2 function decreases *in vitro* parasite infectivity

**Figure 5:**
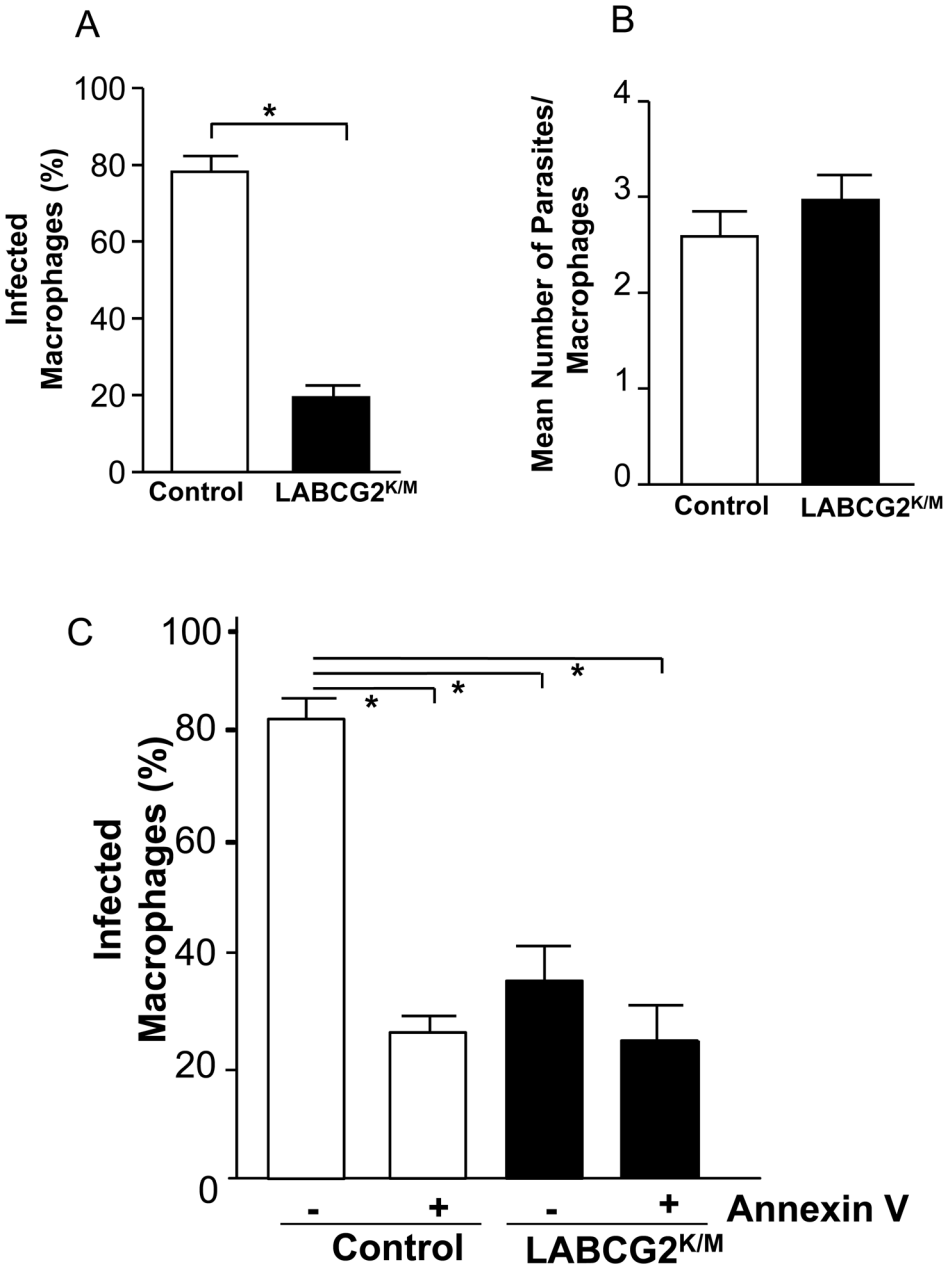
LABCG2^K/M^ parasites are less infective to mouse peritoneal macrophages. Infection of mouse peritoneal macrophages with stationary *Leishmania* promastigotes from control and LABCG2^K/M^ parasites was performed as described in Materials and Methods. The percentage of infected macrophages (A) and the mean number of parasites per macrophage (B) were determined 24 h post-infection. The results represent the means ± SD of three independent experiments. *P<0.05 vs. infection level of control parasites. Additionally, the effect of Annexin V-binding on macrophage infectivity was determined (C) using control and LABCG2^K/M^ stationary parasites incubated in the presence (+) or absence (−) of Annexin V (0.05 µg/µl×10^7^ stationary promastigotes) for 4 h. The results shown are the means of three independent experiments ± SD. *P<0.05 untreated control vs.: Annexin V-treated control, untreated LABCG2^K/M^ parasites and Annexin V-treated LABCG2^K/M^ parasites.

As PS externalization by the parasite is a key mediator for infection of the macrophages [12] and polymorphonuclear cells [16], we evaluated whether down-regulation of LABCG2 function correlated with a decreased infectivity of the parasites. Thus, we measured the ability of control and LABCG2^K/M^ stationary-phase promastigotes to infect mouse peritoneal macrophages. The results showed that whereas 80% of macrophages were infected by control parasites after 24 h post-infection, only 20% of cells were infected by LABCG2^K/M^ parasites (Fig. 5A). In contrast, the number of parasites per infected macrophage was similar in both cases (Fig. 5B). We have shown that the different infectivity values observed in LABCG2^K/M^ parasites are not due to differences in the interaction parasite-macrophages as determined after 4 hours post-infection binding assays (Appendix in Supporting information, Fig. S6A and B. The results showed there were not differences in the percentage of interaction in control cells (80.50±5.77) compared with LABCG2^K/M^ parasites (84.33±7.35). Additionally, we have determined that the overexpression of LABCG2 in *Leishmania* parasites did not show differences in the PS exposition nor in the % infectivity of mouse peritoneal macrophages (data not shown).

We have repeated the infection experiments masking most of the PS in the metacyclic forms by incubating stationary-phase control and LABCG2^K/M^ parasites with Annexin V. As expected, PS masking reduced the macrophage infection percentage of control parasites by approximately 82%. Annexin V-mediated masking of PS in LABCG2^K/M^ parasites did not significantly altered their lower ability to infect peritoneal macrophages, reaching similar values to those obtained with Annexin V treated control parasites (Fig. 5C). Furthermore, we assessed whether other molecules, such as lipophosphoglycan (LPG) or the phosphatidylinositol-anchored surface molecule gp63 [15], both of which are implicated in *Leishmania* infectivity, could be altered in LABCG2^K/M^ parasites. Flow cytometry analysis of stationary-phase promastigotes marked with fluorescein-conjugated ricin agglutinin, which specifically label LPG [45], showed no differences between control and LABCG2^K/M^ parasites (Appendix in Supporting information, Fig. S6C). Additionally, flow cytometry analysis using a specific monoclonal antibody for *Leishmania* gp63 showed no significant differences between expression of this surface molecule in the control and LABCG2^K/M^ stationary-phase promastigotes (Appendix in Supporting information, Fig. S6D). Finally, we evaluated whether the infectivity differences observed may be due to an alteration in the metacyclogenesis of the parasites produced by down-regulation of LABCG2 function. Thus, we purified infective metacyclic forms from stationary-phase promastigotes of control and LABCG2^K/M^ parasites by binding to the lectin peanut agglutinin (PNA) [57], and found that the percentage of metacyclic parasites (PNA^−^) obtained was similar in both cell lines (Appendix in Supporting information, Fig. S7A). Furthermore, both parasite lines were morphologically elongated, highly mobile, there were no rounded shapes and differences in size (FSC-H) between control (427.26±13.82) and LABCG2^K/M^ (413.61±14.53) lines, respectively (Appendix in Supporting information, Fig. S7B). We also analysed expression of the metacyclic marker protein HASPB (hydrophilic acylated surface protein B) [58], which is implicated in host-cell infection. Western blot analysis indicated that there were no differences in expression of this 32 kDa protein between control and LABCG2^K/M^ parasites (Appendix in Supporting information, Fig. S7C).

### LABCG2 is required for disease development in a mouse model of cutaneous leishmaniasis

**Figure 6:**
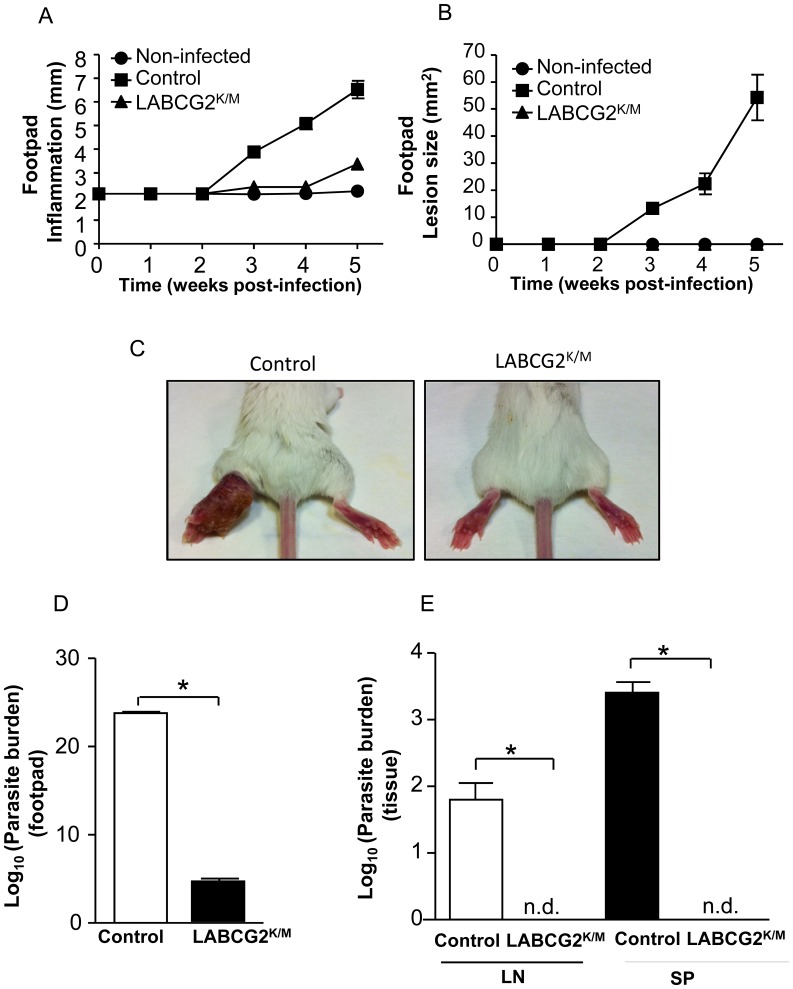
LABCG2^K/M^ parasites are less infective in a cutaneous leishmaniasis mouse model. Susceptible BALB/c mice were infected with 10^4^ control and LABCG2^K/M^ *L. major* metacyclic parasites as described in Materials and Methods. Disease development was monitored weekly by measuring the inflammation (A) and lesion size (B). The pictures in C show the lesion at week 5 post-infection. Parasite burden was determined in footpad (D) and tissues (E): lymph nodes (LN) and spleen (SP). The results represent the means ± SD of three independent experiments, with 10 mice per group. Mice were euthanized when the lesion size in controls reached a value of 50–70 mm^2^. * *P*<0.05 vs. control parasites; n.d. stands for not detected.

As down-regulation of LABCG2 function decreased the *in vitro* macrophage infectivity of metacyclic parasites, we analysed whether this defect was correlated with a lower *in vivo* virulence of the parasites using a mouse model of cutaneous leishmaniasis. Thus, susceptible female BALB/c mice were infected with 10^4^ metacyclics purified from control and LABCG2^K/M^ parasites by footpad inoculation. As we had previously observed during the assays to cure LABCG2^K/M^ parasites for the plasmid pUCNeoplusLABCG2^K/M^ (reverted line) that the defect on the externalization of NBD-PS remained unaltered for at least five weeks in the absence of antibiotic pressure, we were able to use transfected parasites for this model. After infection, the measure of inflammation and the development of skin lesions in the footpad with time were monitored weekly up to a maximum of five weeks. At this time, control animals had to be sacrificed due to the severity of the lesions. Mice infected with control parasites showed progressive inflammation and lesion pathology after the first two weeks (Fig. 6A–C), whereas mice infected with LABCG2^K/M^ parasites showed no detectable lesions pathology at any time, and presented significantly lower footpad inflammation (Fig. 6A–C). As observed in Fig. 6B, the curve for footpad lesion size is the same for non-infected control animals and for the animals infected with LABCG2^K/M^ *L. major* metacyclic parasites, which shows no lesions during the time of the infection assay.

**Figure 7:**
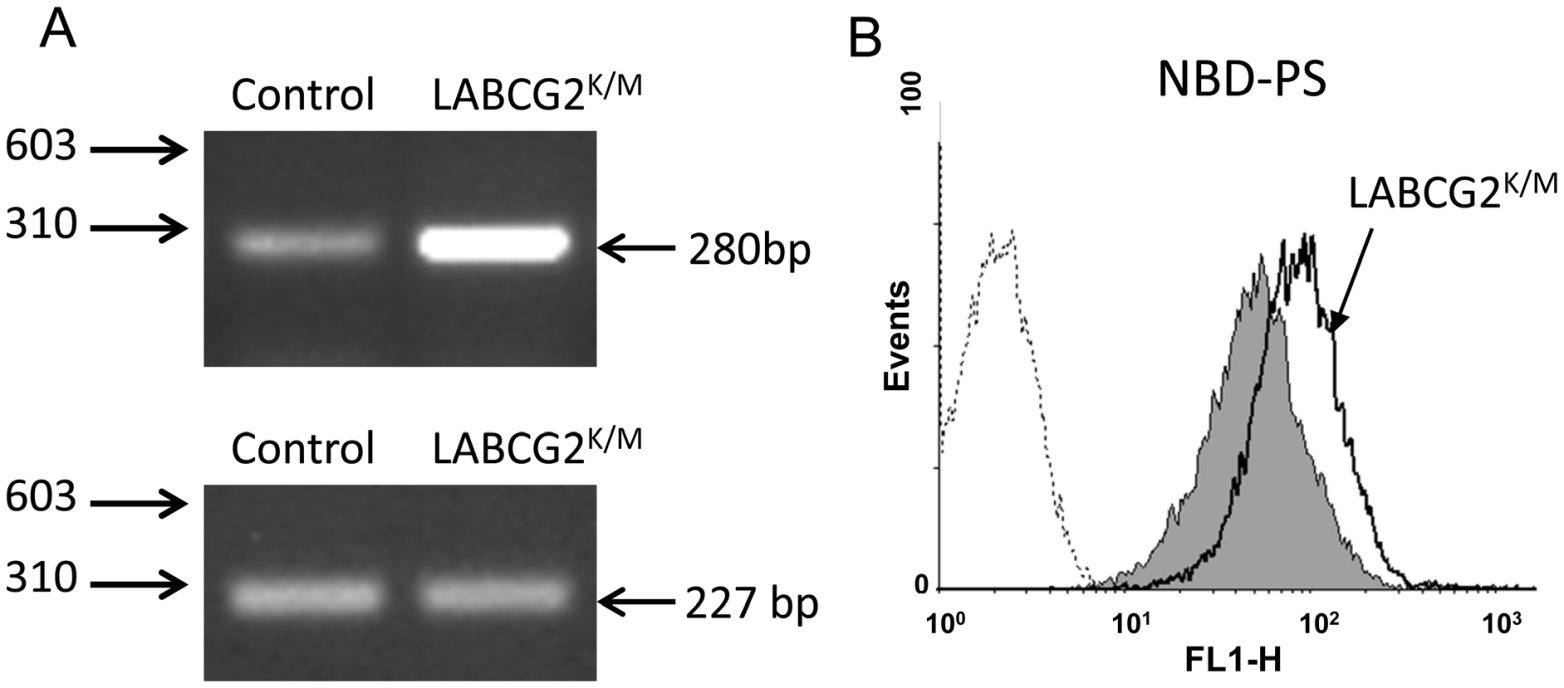
LABCG2^K/M^ parasites isolated from footpad lesions of infected mice retained the increased NBD-PS accumulation phenotype. (A) RT-PCR gene-expression analysis of wild-type and mutated LABCG2 in control and LABCG2^K/M^ *Leishmania* parasites. The lower image shows the expression of GADPH used as internal loading control. RT-PCR was carried out for 35 cycles using RNA isolated from the above parasites and the products were run in 2% agarose gel. The positions of molecular markers (bp) are indicated on the left. (B) Accumulation of NBD-PS in control (grey histogram) and LABCG2^K/M^ (uncoloured histogram) parasites. Parasites recovered from the footpad lesions of infected mice were maintained in culture medium and the stationary promastigotes incubated with the fluorescent phospholipid analogue NBD-PS for 30 min at 28°C. After washing and back-exchange with BSA, cell-associated fluorescence was measured by flow cytometry analysis as described in Materials and Methods. The dotted line represents non-labelled parasites.

Additionally, we decided to determine whether these infected mice presented parasites in different target tissues and whether these parasites maintained the expression of LABCG2^K/M^ and increased NBD-PS accumulation. Thus, at the indicated time, mice were euthanized and their footpad, spleen and lymph nodes collected for parasite isolation following the limiting dilution assay. As can be seen from Fig. 6D–E, a lower parasite burden was recovered from the footpad of mice infected with the LABCG2^K/M^ parasites compared to the control mice (Fig. 6D), and no parasites were isolated from the spleen or lymph nodes of mice infected with the LABCG2^K/M^ parasites (Fig. 6E) after one week of *in vitro* culture. Moreover, we confirmed by RT-PCR that the mutant LABCG2^K/M^ gene was still expressed in parasites isolated from the footpad of mice infected with the LABCG2^K/M^ parasites (Fig. 7A), and that its phenotype of increased NBD-PS accumulation remained unaltered (Fig. 7B). To confirm that the loss of virulence exhibited by the mutant line was due to the expression of LABCG2^K/M^, these experiments were repeated with a second line of LABCG2^K/M^ parasites generated from an independent transfection event. The results of this study were similar to those described above (Appendix in Supporting information, Fig. S8 ), thus indicating that the dramatic differences in the virulence of LABCG2^K/M^ parasites were due to down-regulation of LABCG2 function and suggesting that the *Leishmania LABCG2* gene is crucial for disease development.

## Discussion

The exposure of PS on their cell surface is one of the mechanisms known to be used by *Leishmania* amastigotes and metacyclic promastigotes to infect host macrophages and to inhibit their microbicidal activity [7]–[9], [11], [14], [16]. PS is usually asymmetrically distributed in the cell membrane of eukaryotic cells and is present only in the cytoplasmic leaflet of the plasma membrane [18]. Although the PS synthesis in *Leishmania* have been a matter of intense debate [59], [60], in which the growth state of *Leishmania* parasites could be the possible discrepancy factor, it could be concluded that parasites in late logarithmic phase contain PS.

Although the molecular basis for this outward PS translocation in *Leishmania* remains unknown, herein we provide experimental evidence supporting the involvement of a new ABCG half-transporter (LABCG2) from *Leishmania* in this process.

**Figure 8:**
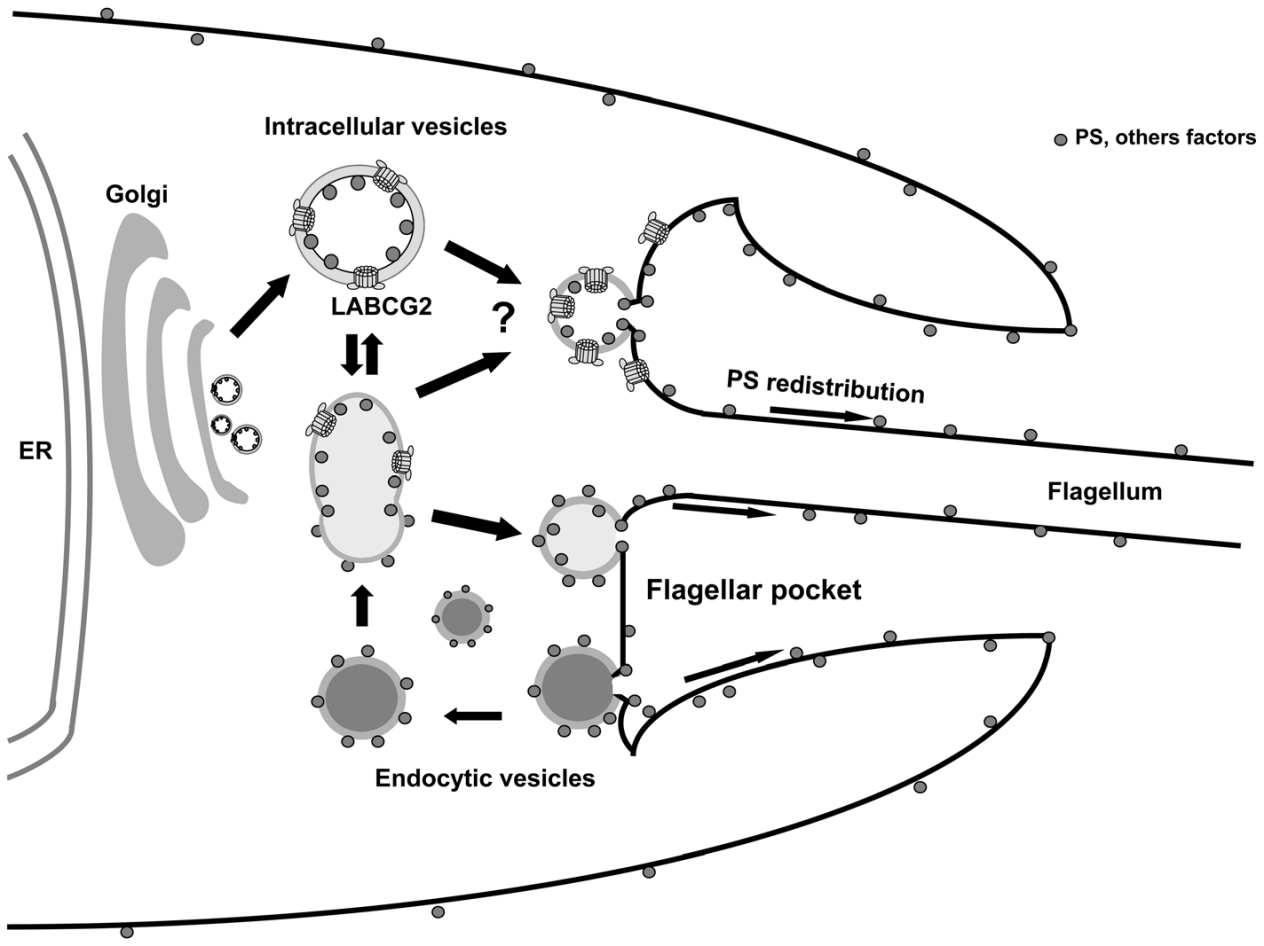
Schematic diagram of the localization of LABCG2 in intracellular vesicles and flagellar pocket of *Leishmania* parasites. It remains possible that LABCG2 could transfer PS and other factors (as virulent factors) to the inner leaflet of intracellular vesicles before their fusion with the plasma membrane at the flagellar pocket, followed by a redistribution of PS/other factors to the outer leaflet of the plasma membrane of the parasite.

Thus, down-regulation of LABCG2 function in *L. major* upon expression of an inactive version of the transporter (LABCG2^K/M^) diminishes the outward translocation of fluorescent short-chain analogues of PS (NBD-PS). This dominant-negative phenotype seems to be specific for the head of PL, as the movement of fluorescent analogues of PC, PE or SM was not affected. This substrate specificity contrasts with the broader spectrum of PL translocated by other lipid floppases [33], [36] and flippases [42] characterized in *Leishmania*. Similar results were obtained after expression of LABCG2^K/M^ in *L. donovani* and *L. infantum*, thereby suggesting that the functionality of LABCG2 could be conserved in *Leishmania* spp. It should be noted, however, that this phenotype was not due to an LABCG2-mediated alteration of PL endocytosis as the internalization of NBD-SM (which is taken up by lipid-phase endocytosis) was not altered. In addition, Annexin V-Alexa 488 binding assays showed that LABCG2^K/M^ promastigotes in the stationary phase exposed less endogenous PS than control parasites. As Annexin V is not entirely specific for PS, despite its widespread use in labelling it, and could bind to other anionic PL [60]–[62], we performed an additional control using papuamide B, a cytolytic peptide that specifically recognizes PS residues in biological membranes [54]. The reduced exposure of PS on the outer face of the plasma membrane in LABCG2^K/M^ parasites was correlated with an increased resistance to papuamide B. Although these differences in resistance to papuamide B were not so higher, such differences were statistically significant and reproducible even in GFP-LABCG2^K/M^ parasites, considering that extreme changes in the phospholipid asymmetry of the eukaryotic membranes lead to cell death [5], [22], [63]. The differences in Annexin V labelling between control and LABCG2^K/M^ parasites were not observed during the log growth-phase of parasites, where the labelling was very low. This finding is in agreement with previous reports for *L. donovani* and *L. tropica* [14] and suggests that LABCG2-mediated PS exposure could be induced during metacyclogenesis of the parasite and is probably maintained in the intracellular amastigote stage. The constitutive expression of LABCG2 in both life-cycle stages of *L. donovani* has been described recently [34]. Although not discussed in detail in that study, LABCG2 expression seems to be higher in the amastigote forms than in the log-phase promastigote forms [34], as would be expected for a protein involved in PS externalization. In our case, the results have shown that the expression of LABCG2 increases in the early and late stationary phase compare with the log phase and this increase would be involved in the redistribution of PS to the outer leaflet of the plasma membrane in the metacyclic forms of the parasite. On the other hand, the localization of LABCG2 at both the flagellar pocket and the aflagellar pole as well as in intracellular vesicles, suggests a possible mechanism for PS exposure in *Leishmania*, similar to that described for human ABCG2, namely as an intracellular sterol transporter [64]. It remains possible that LABCG2 could transfer PS and other factors (as virulent factors) to the inner leaflet of these vesicles before their fusion with the plasma membrane at the flagellar pocket, followed by a redistribution of PS/other factors to the outer leaflet of the plasma membrane of the parasite (Fig. 8). In the case of LABCG2^ K/M^, the content of PS and other factors could be reduced in the intracellular vesicles and in the outer leaflet of plasma membrane, consequently influencing in the infectivity and virulence. A similar situation has been described for the LtrABCA2 transporter of *Leishmania*, involved in phospholipid trafficking and localized at the flagellar pocket and internal vesicles [43]. Additionally, LABCG2 could function as a floppase of PS/other factors due to their localization in the flagellar pocket of the parasite (Fig. 8).

In addition, the defect in PS externalization produced by down-regulation of LABCG2 function was found to be correlated with a significant reduction of the infection of mouse peritoneal macrophages by LABCG2^K/M^ parasites. This finding agrees with previous reports [7], [14], which showed that PS exposure was important for macrophage engulfment of the promastigote and amastigote forms of *Leishmania*. As suggested [12], we believe that the only process probably affected would be the phagocytosis and consequently the variation of PS exposure could not be important for the interaction; this consideration would explain the observed differences in % of infected macrophages. Additionally, the reduced infectivity was not due to changes in the metacyclogenesis process considering that LABCG2 function does not affect either expression of the metacyclic marker protein HASPB or the number of metacyclic promastigotes produced.

Furthermore, the virulent factors LPG and gp63 are probably not affected by the function of LABCG2, as deduced by the absence of variations in the expression levels of these surface molecules, both of which have been suggested that play important roles in establishment of the infection. However, we cannot discard the possibility that other unknown factors involved in the virulence of *Leishmania* could be transported by LABCG2, and their expression could be affected in the LABCG2^K/M^ parasites.

The decrease of the *in vitro* ability to infect macrophages observed in mutant parasites with a defect in PS exposure was found to be correlated with lower infectivity in an *in vivo* mouse model of cutaneous leishmaniasis, thereby supporting the proposal that LABCG2 function is required in order to develop the lesion. The role of the apoptosis-like PS exposure in the establishment of a *Leishmania* infection has been widely discussed. One possibility in this respect is that PS-positive cells do not necessarily die but use PS exposure, in an apoptosis-mimicking fashion, to infect macrophages and inhibit their microbicidal activity [8], [11], [16], [65]. The second possibility suggests that PS-positive forms are indeed “altruistic” apoptotic parasites that have been sentenced to death but are nevertheless required in order that the PS-negative infective parasites invade the host cell, in a truly cooperative system [16], [66]. Should be noted considered that these experiments were made using late stationary phase *Leishmania* parasites, where they begin to appreciate the apoptotic round shapes described above. In our case, the experiments were performed using early stationary phase *Leishmania* parasites, where rounded apoptotic forms were not detected. In any case, PS exposure is the relevant phenotype for macrophages infection and subsequent inactivation of microbicidal activity in both these scenarios, thereby allowing parasite persistence in the host [65].

The need for ABC half-transporters such as ABCG proteins to dimerize in order to reconstitute the ATP-binding sites and become functional has allowed us to test a dominant-negative approach to down-regulate LABCG2 function. Dominant-negative inhibition of mammalian ABCG2 [50], [67] and *Leishmania* LABCG5 [35] has also been described upon expression of different mutants of these transporters. Although the inhibition of homodimeric LABCG2 function is the most likely explanation for expression of the inactive version of LABCG2, we cannot rule out the inhibition of other putative partners. Indeed, some ABCG proteins, such as human ABCG5/8 [68] and *Drosophila* White, Brown and Scarlet, work as heterodimers [69]; the latter can even change the substrate transported as a function of the partner of the White protein. However, as expression of inactive versions of LABCG4 [32] and LABCG5 does not affect the translocation of PS [35] or the infectivity of the parasites, it is more likely that LABCG2 does not heterodimerize with these other *Leishmania* ABCG proteins; it could however dimerize with LABCG1, which is almost identical in its TMD.

A further attractive alternative could be that truncated LABCG3, which only includes the conserved Walker B, the signature motifs and two transmembrane segments and is expressed at least at mRNA levels [31], [34], could dimerize with LABCG2 to produce an inhibitory effect on LABCG2 function. This could be a novel way to regulate protein function in this parasite, although further experiments will be required to confirm this hypothesis.

Finally, LABCG2 belongs to the same subfamily as mammalian ABCG2, a well-characterized PS transporter [30] that also pumps drugs conferring a MDR phenotype in cancer cells [70], [71]. Others LABCG proteins, such as LABCG4 and LABCG6, also confer resistance to leishmanicidal agents [32], [33]. Future work in our group will examine the implication of LABCG2 in drug resistance.

In conclusion, we have provided evidence that strongly suggests the involvement of *Leishmania* LABCG2 function in the PS externalization required for the infection of host macrophages and development of the disease. Nonetheless, new approaches will be needed to fully understand the molecular mechanism by which LABCG2 affects the infectivity and virulence of *Leishmania*. Additionally, these findings indicate that LABCG2 could be considered as a promising drug target for leishmaniasis. Furthermore, as mutant parasites could be isolated from infected mice despite the absence of lesions, null mutants for LABCG2 could potentiality be used for live vaccination studies and to understand the role of LABCG2 in *Leishmania* pathogenesis.

## Supporting Information

Figure S1 **(A) Membrane topology model of the** ***Leishmania*** **half-transporters LABCG1, LABCG2 and LABCG3**. The nucleotide binding domain (NBD) is located N-terminal with respect to the transmembrane domain (TMD). The putative membrane-spanning helices of the TMD are shown as cylinders passing through the lipid bilayer. The ATPase catalytic Walker A, Walker B, and the signature motif C localized in the nucleotide binding domain (NBD) are shown (boxes A, B and C, respectively). The arrow indicates the catalytic site mutation (K108M) engineered into the Walker A motif. The topology model was predicted using TMHMM (http://www.cbs.dtu.dk/services/TMHMM-2.0/) and TMRPres2D ((http://biophysics.biol.uoa.gr/TMRPres2D/) softwares. **(B) Amino acid sequences and alignment (Clustal W) of** ***L. major*** **LABCG1, LABCG2 and LABCG3**. Putative transmembrane segments predicted by TMHMM and TMRPres2D are underlined. The Walker A/Walker B motifs, and the ABC family signature motif C are boxed. Identical amino acids present in the three sequences are indicated by *, the amino acid similarity is indicated by : and weak similar amino acid are indicated by **^.^** . Gaps introduced for the sequence alignment are indicated by -.
(TIF)

Figure S2 **Gene-expression analysis of LABCG2 in** ***Leishmania*** **lines.** (A) Upper panel: gene expression of LABCG2 by RT-PCR as indicated by the amplified 280 bp *LABCG2* fragment. Lower panel: gene expression of GADPH as internal loading control. The arrow indicates amplified 227 bp GADPH fragment. Lane 1: DNA marker phi 174 *Hae*III; lane 2: control parasites; lane 3: LABCG2^K/M^ parasites. RT-PCR was carried out for 35 cycles using RNA isolated from the above-mentioned parasites and the products run in 2% agarose gel. (B) Growth curve of control (black circles) and LABCG2^K/M^ (black squares) parasites at different time points (24, 48, 72, 96 and 120 h). The results represent the means ± SD of three independent experiments.
(TIF)

Figure S3 **Protein expression in GFP-LABCG2 and GFP-LABCG2^K/M^** ***Leishmania*** **parasites.** Immunodetection of GFP (A) or H2A histone (*lower inset*) in *L. major* lines expressing control GFP (lane 1), GFP-LABCG2 (lane 2) and GFP-LABCG2^K/M^ (lane 3). Western blot analysis of total proteins from parasites incubated with antibodies against GFP or H2A histone, as loading control, at a 1∶5000 dilution. The molecular mass standards (kDa) from Bio-Rad are indicated on the left. (B) *L. major* stationary promastigotes transfected with pXG-GFP+ and LABCG2^K/M^-GFP were fixed for 10 min in 2% paraformaldehyde at 4°C. *a* and *c*, Nomarski images of *b* and *d*, respectively. *b* shows the cytoplasmic localization of the protein GFP and *d* corresponds to localization sites of LABCG2^K/M^-GFP, indicated by white arrows in the merged images. Scale bar: 5 µm. FP: flagellar pocket; AP: aflagellar pole. The figure illustrates a representative parasite of a total population of parasites with a similar fluorescence pattern.
(TIF)

Figure S4 **Fluorescent PS accumulation in** ***Leishmania*** **parasites.** Stationary promastigotes of *L. infantum* (A) or *L. donovani* (B) were incubated with the fluorescent PL analogue NBD-PS for 30 min at 28°C. After washing and back-exchange with BSA, cell-associated fluorescence was measured by flow cytometry analysis. The grey histogram represents control parasites transfected with the empty vector, the uncoloured histogram represents parasites expressing LABCG2^K/M^ and the dotted histogram represents non-labelled cells. The histograms correspond to a representative experiment from three independent experiments.
(TIF)

Figure S5 **The externalization of endogenous PS in** ***Leishmania*** **parasites.** PS exposure at the outer leaflet of the parasite plasma membrane was analyzed by flow cytometry using Annexin V–Alexa 488 in control parasites as described in Materials and Methods. Controls measurements in the absence of calcium were included using Annexin V–Alexa 488 plus 8 mM EGTA. The results shown are representative of three independent duplicated experiments ± SD. * *P*<0.05 vs. control parasites.
(TIF)

Figure S6 **LABCG2^K/M^ parasites has not affected its capacity of binding to macrophages.** (A) Binding of control and LABCG2^K/M^ metacyclic promastigotes to mouse peritoneal macrophages. Percentage of positive interaction of promastigotes to macrophages after 4 h interaction was determined by a fluorescence microscopy analysis counting 100 macrophages/well. The results represent the means ± SD of three independent experiments. (B) Micrograph of double-fluorescence labeling of the binding of *Leishmania* control and LABCG2^K/M^ metacyclic parasites to mouse peritoneal macrophages. Cell Tracker TM Green-labeled parasites were added (5∶1) to mouse peritoneal macrophages relabeled with FM4-64 (red). *a* and *c*, Nomarski images of *b* and *d*, respectively. *b* and *d* shows the binding and intracellular localization of control and LABCG2^K/M^ parasites. Scale bar: 10 µm. The expression levels of two surface molecules, LPG (C) and gp63 (D), were determined in control and LABCG2^K/M^ parasites marked with fluorescein-conjugated ricin agglutinin that specifically labels LPG (C) and a specific monoclonal antibody for *Leishmania* gp63 (D). The fluorescence intensity was determined by flow cytometry analysis, as described in Materials and Methods. The data are means of the geometrical mean channel fluorescence values (g.m.) ± SD of three independent experiments versus controls.
(TIF)

Figure S7 **LABCG2^K/M^** ***Leishmania*** **parasites do not show defective metacyclogenesis.** (A) Control and LABCG2^K/M^ metacyclic parasites were purified from stationary promastigotes using negative selection with the lectin PNA, as described in Materials and Methods. The results represent the means ± SD of four independent experiments. (B) Analysis by flow cytometry of the FSC-H of control and LABCG2^K/M^ metacyclic parasites; right panel shows a Nomarsky micrography of the same samples. Scale bar: 5 µm. (C) Total cell lysates from stationary promastigotes were analyzed by Western blotting with an antibody to the metacyclic protein HASPB. Anti-alpha tubulin antibody was used as loading control. The positions of molecular marker (kDa) are indicated on the left.
(TIF)

Figure S8 **LABCG2^K/M^ parasites are less infective in a mouse model of cutaneous leishmaniasis.** A second independent transfection event using control and LABCG2^K/M^ parasites was inoculated in mice, as described in Materials and Methods. The inflammation (A), lesion size (B) and parasite burden in footpad (D) and tissues (E) such as lymph nodes (LN) and spleen (SP) were determined weekly. The pictures in C show the lesion at week 5 post-infection. The results represent the means ± SD of three independent experiments, with 10 mice per group. Mice were euthanized when the lesion size in controls reached a value of 50–70 mm^2^. *P<0.05 vs. control parasites; n.d. stands for not detected.
(TIF)
