# Correction: LABCG2, a New ABC Transporter Implicated in Phosphatidylserine Exposure, Is Involved in the Infectivity and Pathogenicity of *Leishmania*

**DOI:** 10.1371/annotation/6a3b1d53-4e80-45a9-8fab-f3fb56a134de

**Published:** 2013-06-12

**Authors:** Jenny Campos-Salinas, David León-Guerrero, Elena González-Rey, Mario Delgado, Santiago Castanys, José M. Pérez-Victoria, Francisco Gamarro

José M. Pérez-Victoria should be noted as co-corresponding author/primary investigator with Francisco Gamarro. His email address is josepv@ipb.csic.es

