# Supplementary figures and images for: LABCG2, a New ABC Transporter Implicated in Phosphatidylserine Exposure, Is Involved in the Infectivity and Pathogenicity of Leishmania
Source: PLoS Negl Trop Dis. 2013 Apr 25;7(4):e2179. doi: 10.1371/journal.pntd.0002179 (PMC3636091; doi:10.1371/journal.pntd.0002179)

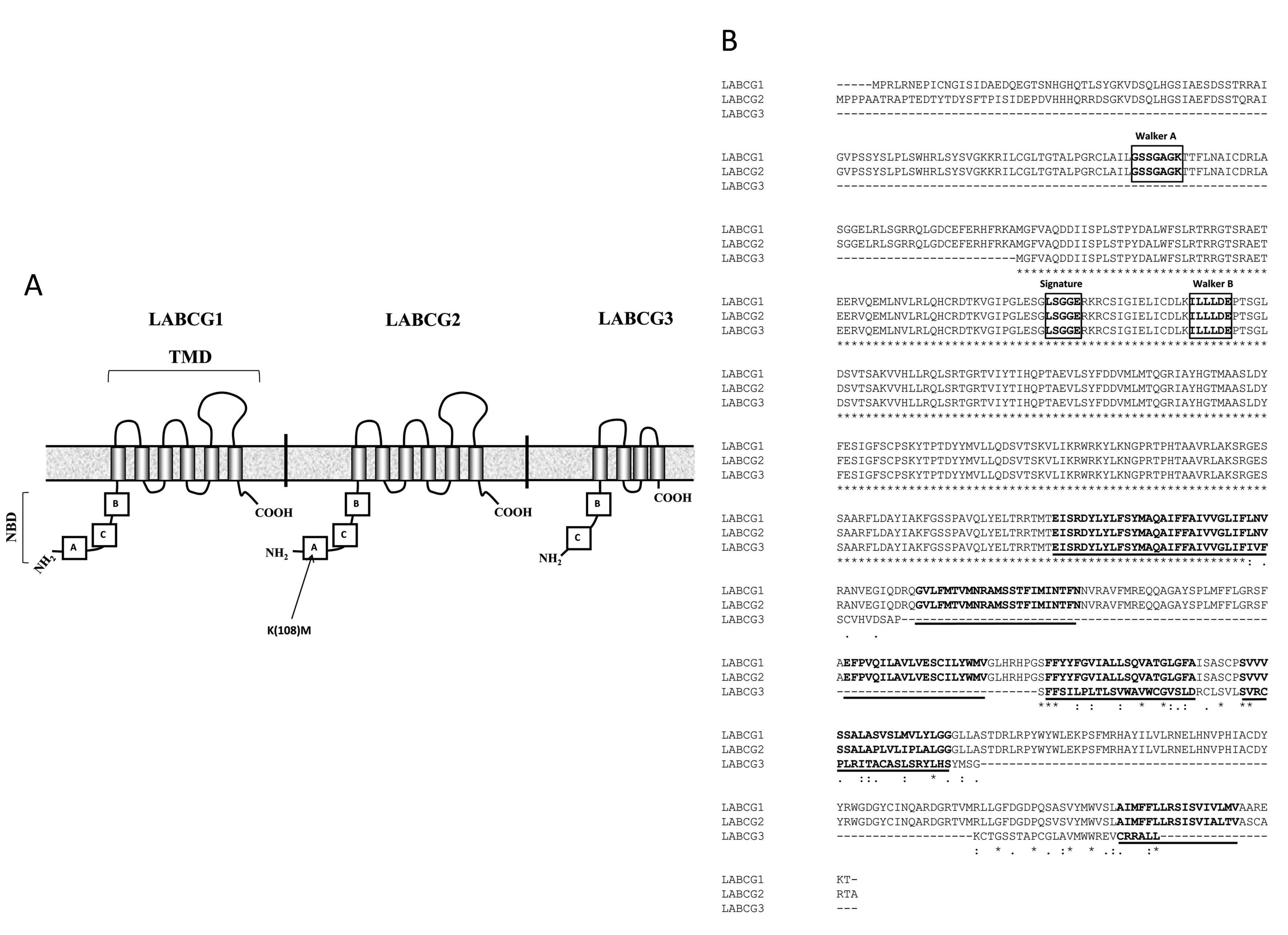

Supplement: Figure S1 — (A) Membrane topology model of the Leishmania half-transporters LABCG1, LABCG2 and LABCG3. The nucleotide binding domain (NBD) is located N-terminal with respect to the transmembrane domain (TMD). The putative membrane-spanning helices of the TMD are shown as cylinders passing through the lipid bilayer. The ATPase catalytic Walker A, Walker B, and the signature motif C localized in the nucleotide binding domain (NBD) are shown (boxes A, B and C, respectively). The arrow indicates the catalytic site mutation (K108M) engineered into the Walker A motif. The topology model was predicted using TMHMM (http://www.cbs.dtu.dk/services/TMHMM-2.0/) and TMRPres2D ((http://biophysics.biol.uoa.gr/TMRPres2D/) softwares. (B) Amino acid sequences and alignment (Clustal W) of L. major LABCG1, LABCG2 and LABCG3. Putative transmembrane segments predicted by TMHMM and TMRPres2D are underlined. The Walker A/Walker B motifs, and the ABC family signature motif C are boxed. Identical amino acids present in the three sequences are indicated by *, the amino acid similarity is indicated by : and weak similar amino acid are indicated by . . Gaps introduced for the sequence alignment are indicated by -. (TIF) [file pntd.0002179.s001.tif]

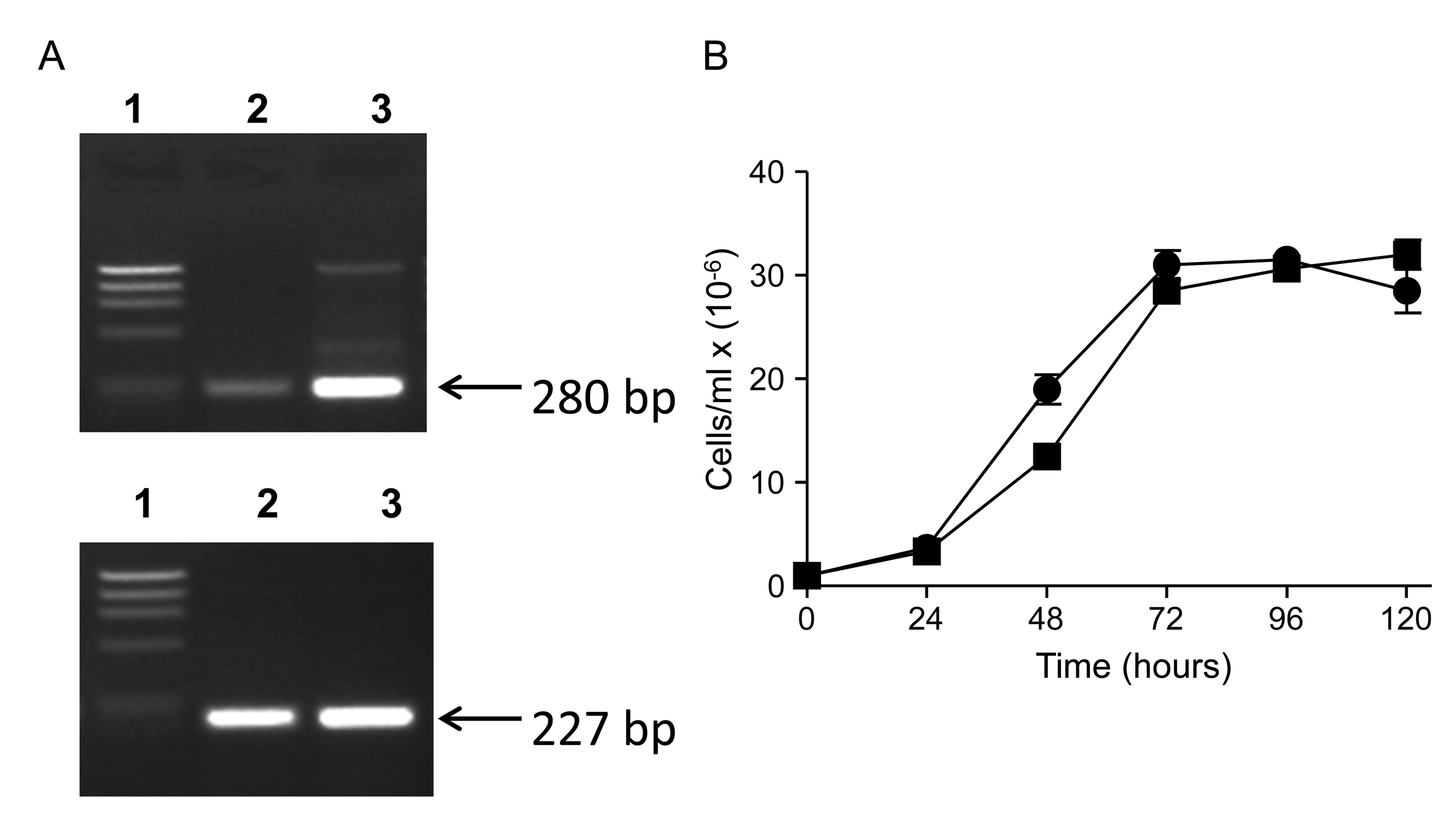

Supplement: Figure S2 — Gene-expression analysis of LABCG2 in Leishmania lines. (A) Upper panel: gene expression of LABCG2 by RT-PCR as indicated by the amplified 280 bp LABCG2 fragment. Lower panel: gene expression of GADPH as internal loading control. The arrow indicates amplified 227 bp GADPH fragment. Lane 1: DNA marker phi 174 HaeIII; lane 2: control parasites; lane 3: LABCG2K/M parasites. RT-PCR was carried out for 35 cycles using RNA isolated from the above-mentioned parasites and the products run in 2% agarose gel. (B) Growth curve of control (black circles) and LABCG2K/M (black squares) parasites at different time points (24, 48, 72, 96 and 120 h). The results represent the means ± SD of three independent experiments. (TIF) [file pntd.0002179.s002.tif]

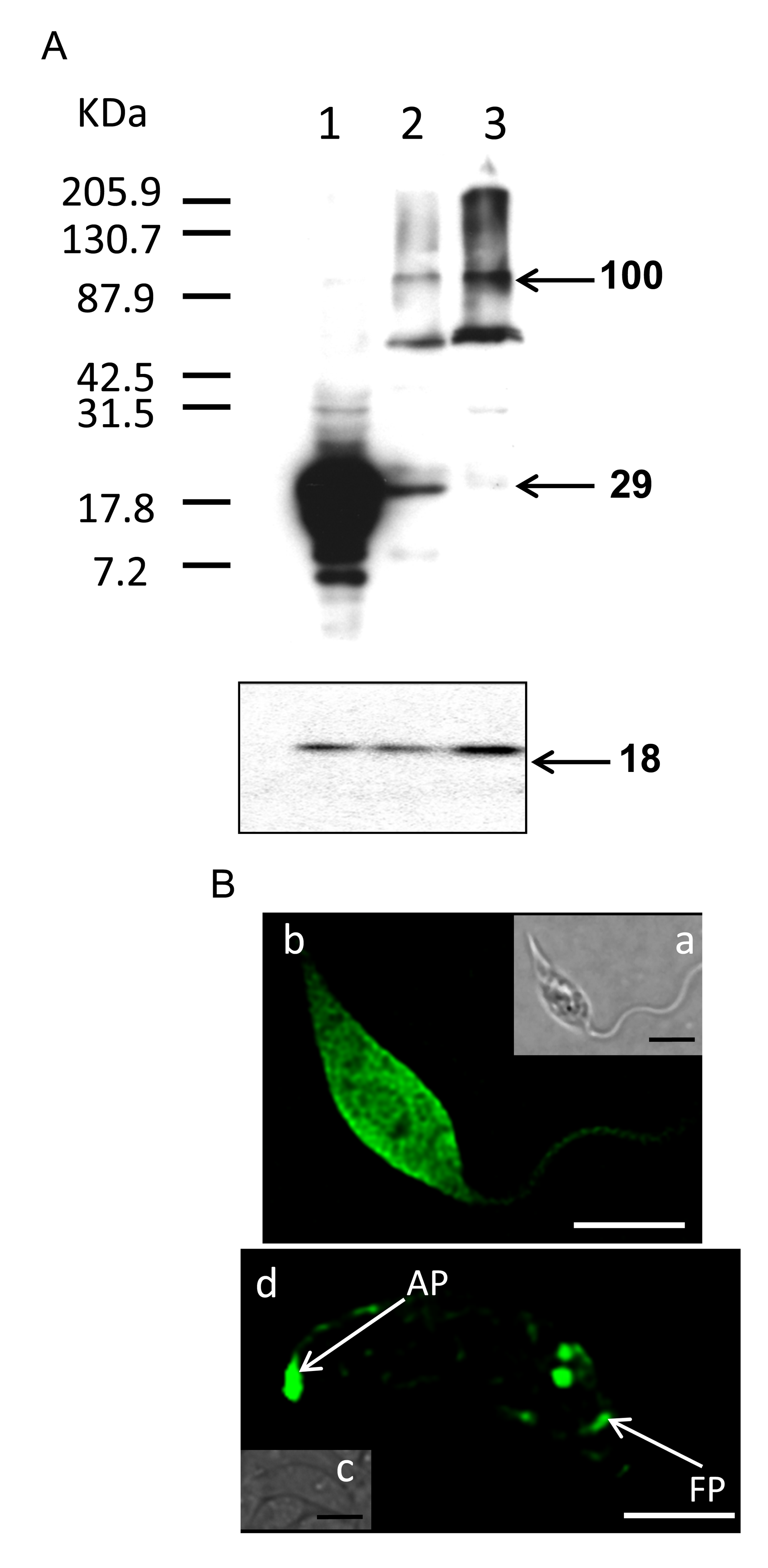

Supplement: Figure S3 — Protein expression in GFP-LABCG2 and GFP-LABCG2K/M Leishmania parasites. Immunodetection of GFP (A) or H2A histone (lower inset) in L. major lines expressing control GFP (lane 1), GFP-LABCG2 (lane 2) and GFP-LABCG2K/M (lane 3). Western blot analysis of total proteins from parasites incubated with antibodies against GFP or H2A histone, as loading control, at a 1∶5000 dilution. The molecular mass standards (kDa) from Bio-Rad are indicated on the left. (B) L. major stationary promastigotes transfected with pXG-GFP+ and LABCG2K/M-GFP were fixed for 10 min in 2% paraformaldehyde at 4°C. a and c, Nomarski images of b and d, respectively. b shows the cytoplasmic localization of the protein GFP and d corresponds to localization sites of LABCG2K/M-GFP, indicated by white arrows in the merged images. Scale bar: 5 µm. FP: flagellar pocket; AP: aflagellar pole. The figure illustrates a representative parasite of a total population of parasites with a similar fluorescence pattern. (TIF) [file pntd.0002179.s003.tif]

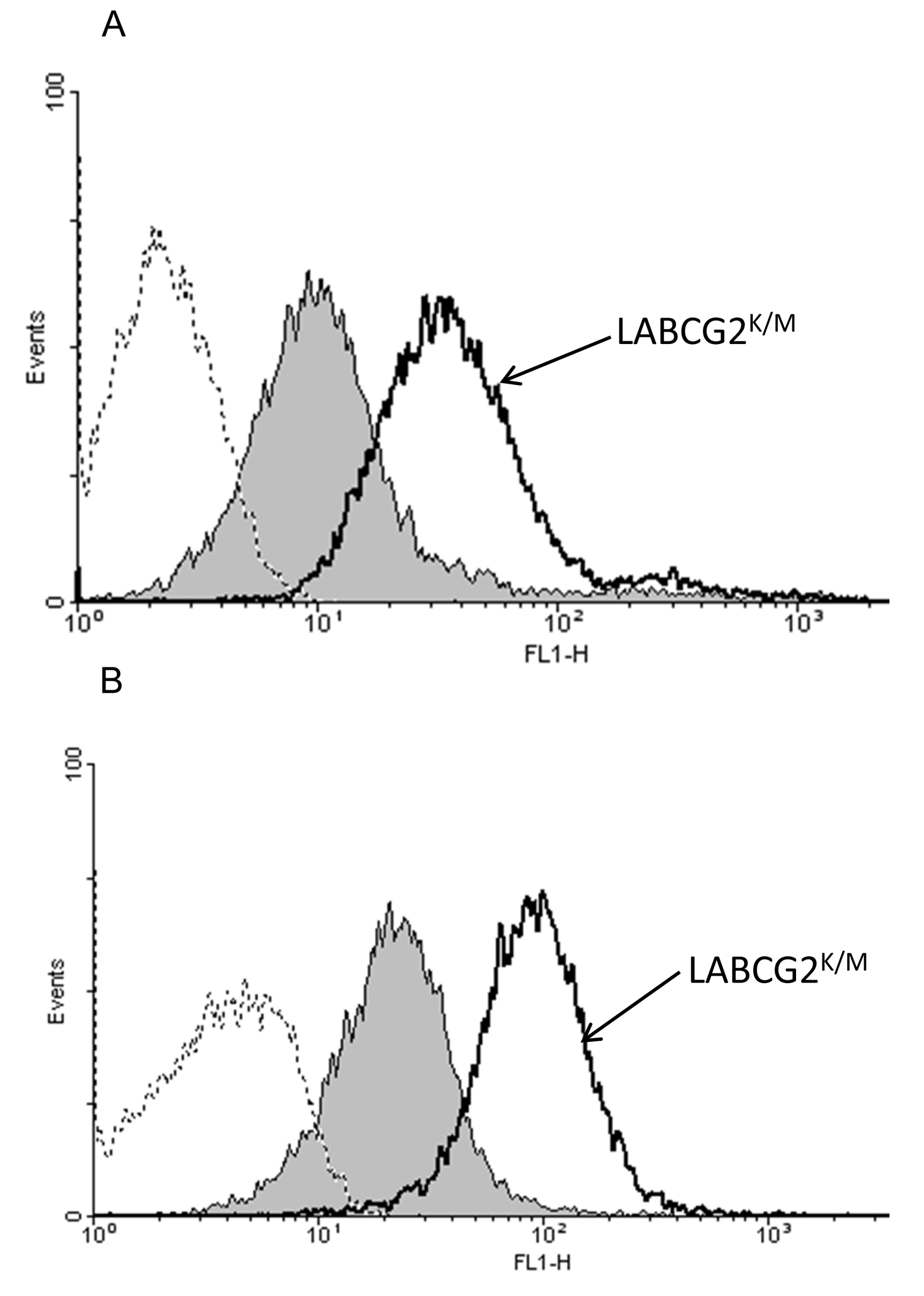

Supplement: Figure S4 — Fluorescent PS accumulation in Leishmania parasites. Stationary promastigotes of L. infantum (A) or L. donovani (B) were incubated with the fluorescent PL analogue NBD-PS for 30 min at 28°C. After washing and back-exchange with BSA, cell-associated fluorescence was measured by flow cytometry analysis. The grey histogram represents control parasites transfected with the empty vector, the uncoloured histogram represents parasites expressing LABCG2K/M and the dotted histogram represents non-labelled cells. The histograms correspond to a representative experiment from three independent experiments. (TIF) [file pntd.0002179.s004.tif]

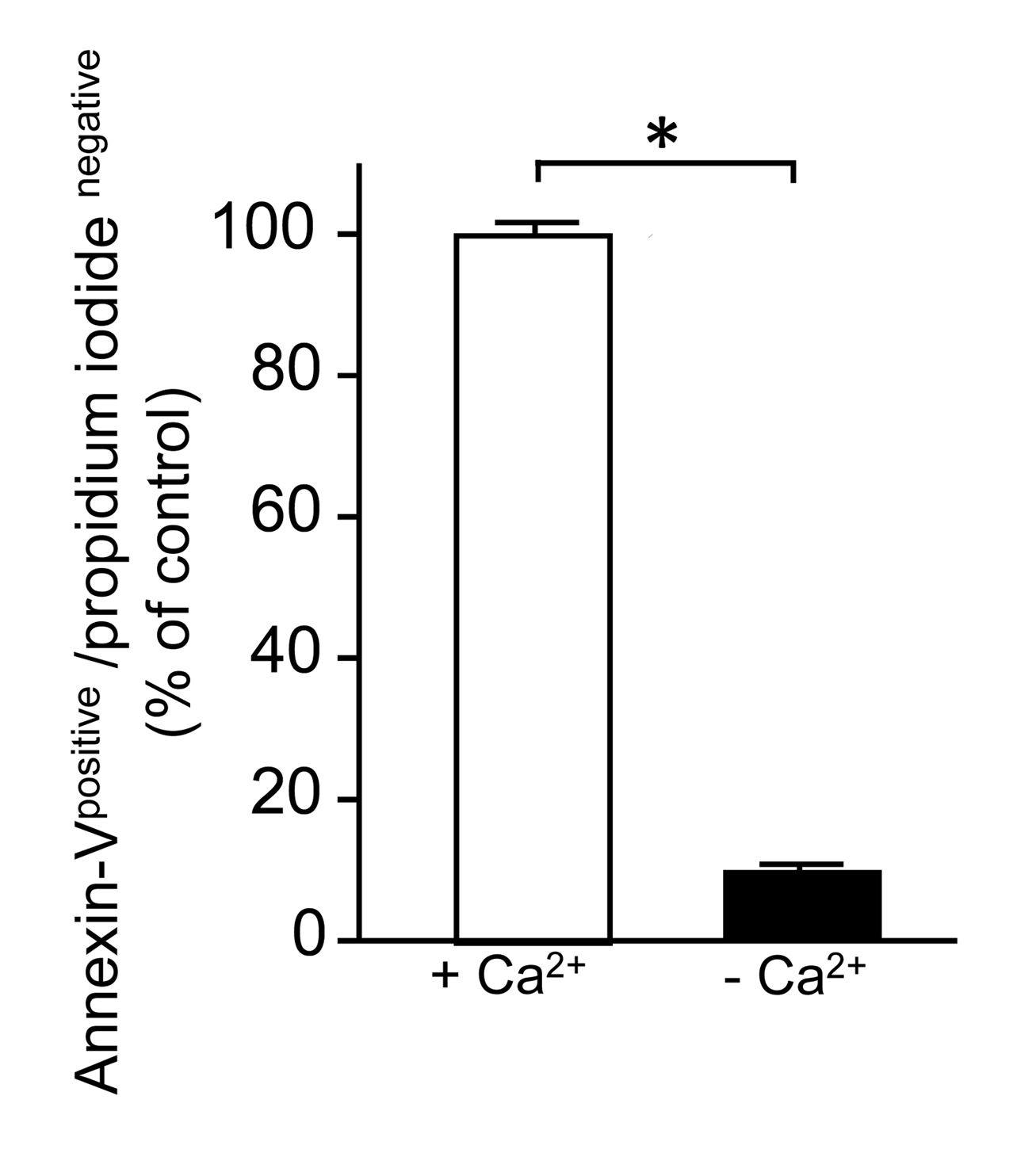

Supplement: Figure S5 — The externalization of endogenous PS in Leishmania parasites. PS exposure at the outer leaflet of the parasite plasma membrane was analyzed by flow cytometry using Annexin V–Alexa 488 in control parasites as described in Materials and Methods. Controls measurements in the absence of calcium were included using Annexin V–Alexa 488 plus 8 mM EGTA. The results shown are representative of three independent duplicated experiments ± SD. * P<0.05 vs. control parasites. (TIF) [file pntd.0002179.s005.tif]

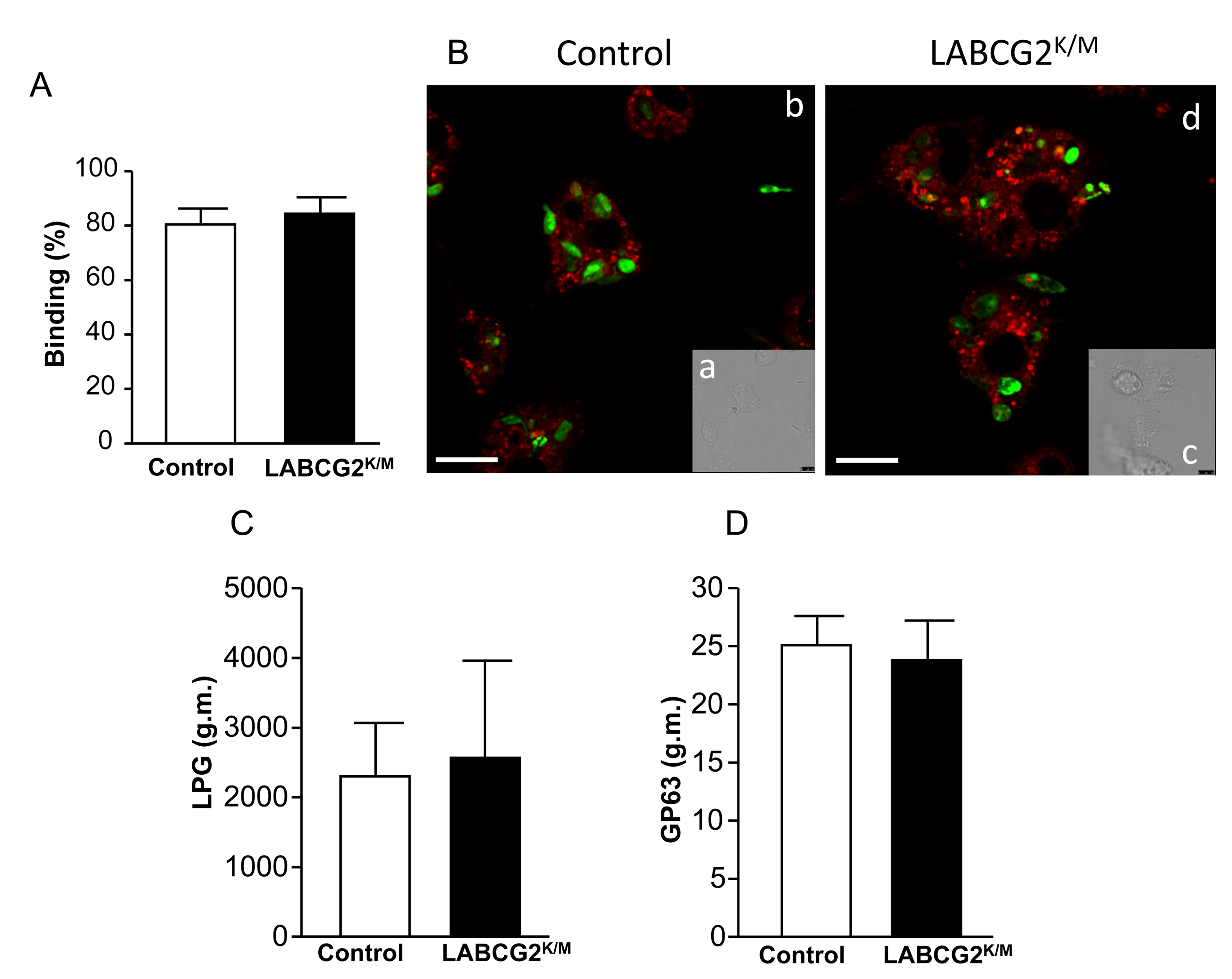

Supplement: Figure S6 — LABCG2K/M parasites has not affected its capacity of binding to macrophages. (A) Binding of control and LABCG2K/M metacyclic promastigotes to mouse peritoneal macrophages. Percentage of positive interaction of promastigotes to macrophages after 4 h interaction was determined by a fluorescence microscopy analysis counting 100 macrophages/well. The results represent the means ± SD of three independent experiments. (B) Micrograph of double-fluorescence labeling of the binding of Leishmania control and LABCG2K/M metacyclic parasites to mouse peritoneal macrophages. Cell Tracker TM Green-labeled parasites were added (5∶1) to mouse peritoneal macrophages relabeled with FM4-64 (red). a and c, Nomarski images of b and d, respectively. b and d shows the binding and intracellular localization of control and LABCG2K/M parasites. Scale bar: 10 µm. The expression levels of two surface molecules, LPG (C) and gp63 (D), were determined in control and LABCG2K/M parasites marked with fluorescein-conjugated ricin agglutinin that specifically labels LPG (C) and a specific monoclonal antibody for Leishmania gp63 (D). The fluorescence intensity was determined by flow cytometry analysis, as described in Materials and Methods. The data are means of the geometrical mean channel fluorescence values (g.m.) ± SD of three independent experiments versus controls. (TIF) [file pntd.0002179.s006.tif]

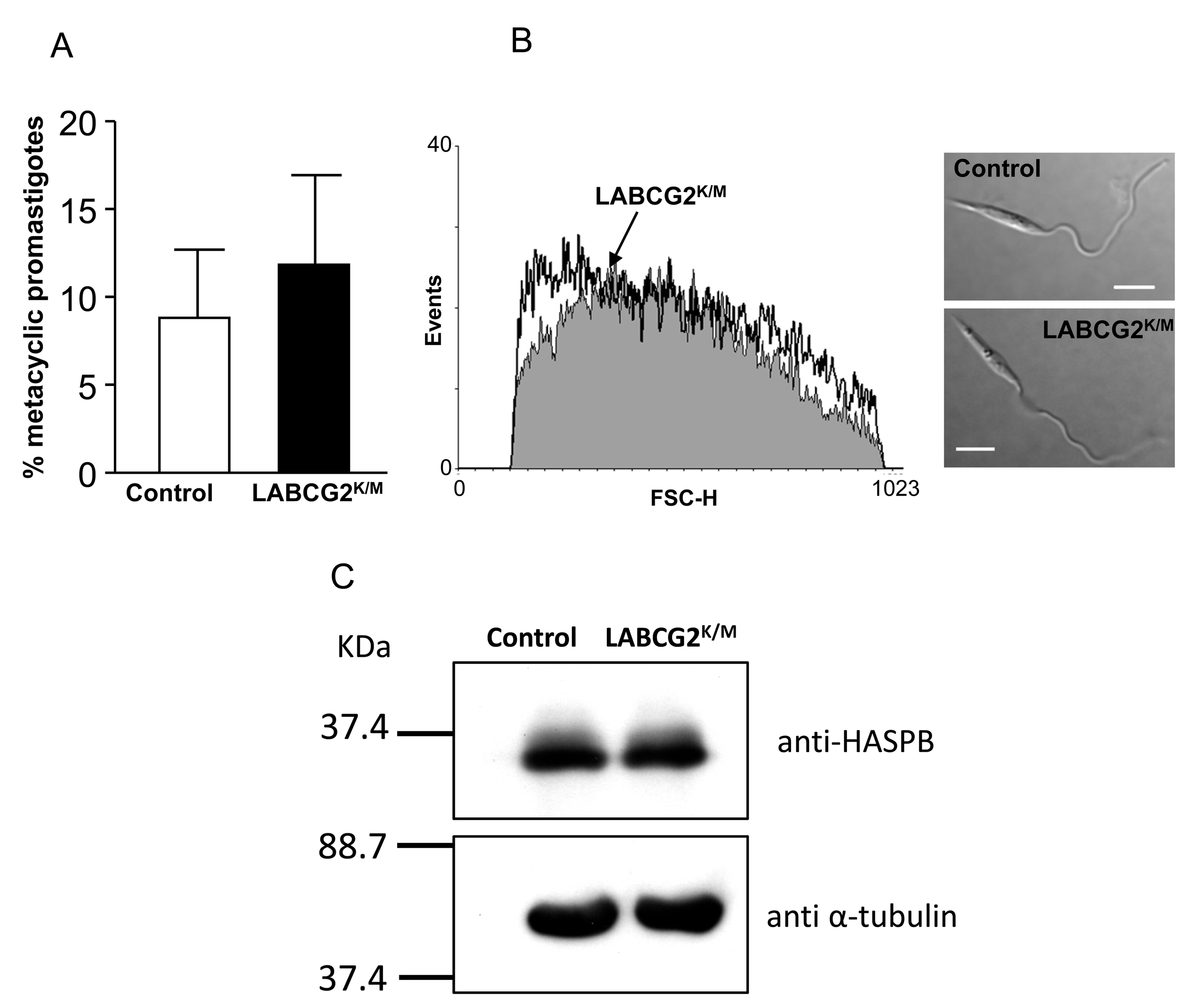

Supplement: Figure S7 — LABCG2K/M Leishmania parasites do not show defective metacyclogenesis. (A) Control and LABCG2K/M metacyclic parasites were purified from stationary promastigotes using negative selection with the lectin PNA, as described in Materials and Methods. The results represent the means ± SD of four independent experiments. (B) Analysis by flow cytometry of the FSC-H of control and LABCG2K/M metacyclic parasites; right panel shows a Nomarsky micrography of the same samples. Scale bar: 5 µm. (C) Total cell lysates from stationary promastigotes were analyzed by Western blotting with an antibody to the metacyclic protein HASPB. Anti-alpha tubulin antibody was used as loading control. The positions of molecular marker (kDa) are indicated on the left. (TIF) [file pntd.0002179.s007.tif]

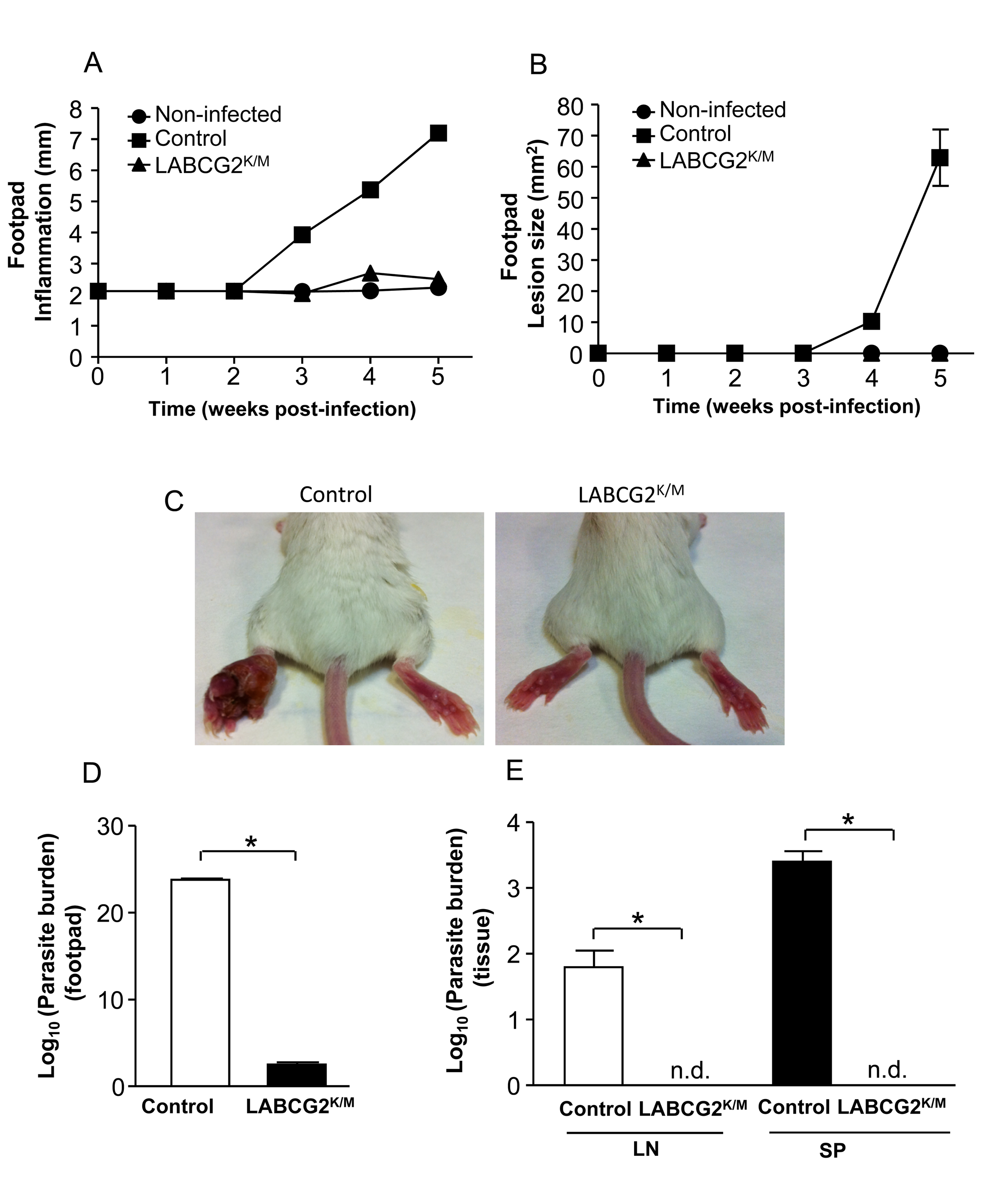

Supplement: Figure S8 — LABCG2K/M parasites are less infective in a mouse model of cutaneous leishmaniasis. A second independent transfection event using control and LABCG2K/M parasites was inoculated in mice, as described in Materials and Methods. The inflammation (A), lesion size (B) and parasite burden in footpad (D) and tissues (E) such as lymph nodes (LN) and spleen (SP) were determined weekly. The pictures in C show the lesion at week 5 post-infection. The results represent the means ± SD of three independent experiments, with 10 mice per group. Mice were euthanized when the lesion size in controls reached a value of 50–70 mm2. *P<0.05 vs. control parasites; n.d. stands for not detected. (TIF) [file pntd.0002179.s008.tif]
